# Prime and Boost Vaccination Elicit a Distinct Innate Myeloid Cell Immune Response

**DOI:** 10.1038/s41598-018-21222-2

**Published:** 2018-02-15

**Authors:** Jean-Louis Palgen, Nicolas Tchitchek, Jamila Elhmouzi-Younes, Simon Delandre, Inana Namet, Pierre Rosenbaum, Nathalie Dereuddre-Bosquet, Frédéric Martinon, Antonio Cosma, Yves Lévy, Roger Le Grand, Anne-Sophie Beignon

**Affiliations:** 1 CEA – Université Paris Sud 11 – INSERM U1184, Immunology of Viral Infections and Autoimmune Diseases, IDMIT department, IBFJ, 92265 Fontenay-aux-Roses, France; 2 Vaccine Research Institute, Henri Mondor Hospital, 94010 Créteil, France; 3 Institut Mondor de Recherche Biomédicale – INSERM U955, équipe 16 physiopathologie et immunothérapies dans l’infection VIH, 94010 Créteil, France

## Abstract

Understanding the innate immune response to vaccination is critical in vaccine design. Here, we studied blood innate myeloid cells after first and second immunization of cynomolgus macaques with the modified vaccinia virus Ankara. The inflammation at the injection site was moderate and resolved faster after the boost. The blood concentration of inflammation markers increased after both injections but was lower after the boost. The numbers of neutrophils, monocytes, and dendritic cells were transiently affected by vaccination, but without any major difference between prime and boost. However, phenotyping deeper those cells with mass cytometry unveiled their high phenotypic diversity with subsets responding differently after each injection, some enriched only after the primary injection and others only after the boost. Actually, the composition in subphenotype already differed just before the boost as compared to just before the prime. Multivariate analysis identified the key features that contributed to these differences. Cell subpopulations best characterizing the post-boost response were more activated, with a stronger expression of markers involved in phagocytosis, antigen presentation, costimulation, chemotaxis, and inflammation. This study revisits innate immunity by demonstrating that, like adaptive immunity, innate myeloid responses differ after one or two immunizations.

## Introduction

Many biological mechanisms involved with vaccination are still unclear and require further characterization. Several studies have highlighted the modulation of adaptive immunity by early innate immunity^1^, which may provide biomarkers to predict immune memory. Deciphering the mechanisms of the early innate immune response to vaccines will be valuable for optimizing them for protective immunity.

Innate myeloid cells are composed of mononuclear phagocytes, monocytes and dendritic cells (DCs), and granulocytes. They are involved in pathogen clearance, induction and resolution of inflammation, and antigen presentation^2,3^. They are often believed to react similarly to the first and subsequent pathogen encounters. Indeed, these cells are activated by germline-encoded pattern recognition receptors (PRR), are short-lived, except macrophages, and unlikely to show memory features^4^. However, enhanced responsiveness to pathogen re-encounter, called trained immunity and related to epigenetic modifications, was reported for monocytes and macrophages^5^. The overall immune status also differs between the first and second pathogen encounters due to the presence of memory B and T cells and antibodies at the second encounter. In particular, antigen-antibody complexes are known to affect innate responses through the interaction of antibodies with Fc receptors found in most innate cells including granulocytes, monocytes/macrophages, and DCs^6,7^. This likely affects the behaviour of innate immune cells. However, this crosstalk between innate and adaptive immunity in the context of repeated vaccine injections, called prime-boost vaccine strategies, is still poorly understood, although they are widely used to increase the frequency of responders and enhance the immunogenicity and protective efficacy of vaccines^8^.

We studied the impact of vaccination on innate myeloid cells by immunizing cynomolgus macaques, which represent a relevant species for human vaccine research^9,10^. We used the modified Vaccinia virus Ankara (MVA), a smallpox vaccine, as a vaccine model to induce robust cellular and humoral immunity^11^. Unlike the vaccinia virus (VACV) from which it was derived, MVA requires a two-dose regimen to induce a strong antibody response and provide full protection against VACV challenge in humans^12,13^ or monkeypox challenge in non-human primates^14^. MVA is also a potent vaccine vector currently being developed against several infectious diseases and cancers^15^. Many studies on cell tropism, innate immune activation and immune evasion used *in vitro* models^16^. However, a comprehensive overview of the mechanisms of MVA-induced immunity *in vivo* is still lacking.

Here, we developed a mass cytometry panel, focusing on innate myeloid cells, with the aim of identifying cell subphenotypes altered by vaccination. Mass cytometry is a promising technology for discovering cell subsets. It can unravel new mechanisms of the immunization process and help to design new vaccines. Currently, longitudinal mass cytometry data analyses following immunization are scarce^17,18^. One of the main analysis challenge is the lack of appropriate pipelines. Here, we used the SPADE algorithm^19^ together with SPADEVizR^20^ to analyze our high-dimensional cytometry data. This analysis pipeline can be used for any kind of multidimensional cytometry data analysis, beyond the study of the dynamic of vaccine-induced immune responses or the study of vaccines modes of action. To make these complex data fully accessible to the scientific community, we publicly released them on the FlowRepository database and the Cytobank platform. We also created a website with interactive representations. We provide evidence of the phenotypic diversity of innate myeloid cells and of the qualitative and quantitative differences in their recruitment following MVA prime-boost immunization. This work constitutes the basis for future studies aiming to decipher how the differences in innate responses after one or two vaccine encounters depend on primary memory responses, and conversely how they affect the development of secondary memory responses.

## Results

### Changes in local and systemic inflammation following MVA prime and boost

**Figure 1:**
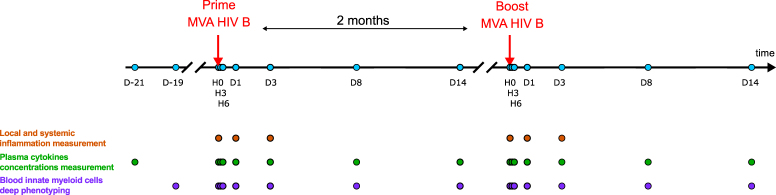
Experimental design and analysis pipeline. Five adult cynomolgus macaques were immunized, two months apart with MVA HIV-B at a dose of 4 × 10^8^ PFU injected subcutaneously. Blood was collected longitudinally at the indicated timepoints, hours (H) or days (D), post-prime (PP) and post-boost (PB), for Luminex, ELISA, and mass cytometry analyses to evaluate the plasma concentrations of cytokines and CRP and to phenotype in deep innate myeloid cells. Local inflammation was also scored at the indicated timepoints. Baseline samples were collected 21 and 19 days before the first vaccine injection for plasma soluble factors and single cell mass cytometry analysis, respectively, as well as just before the first immunization at H0PP. A blood draw was also collected just before the boost at D58PP/H0PB.

**Figure 2:**
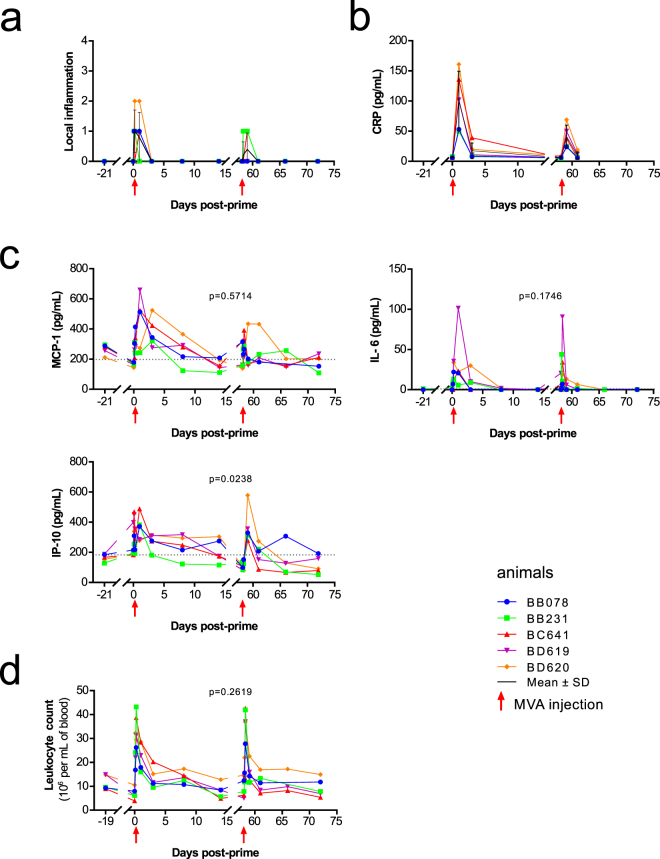
Local inflammation, systemic inflammation and complete blood count. (**a**) Individual local inflammation scores, as well as mean ± standard deviation, are represented over time. Local skin reactions at the site of the subcutaneous MVA injection were scored from 0 to 4, based on the evaluation of edema and erythema. 0: no swelling and normal color; 1: slight swelling with indistinct border and light pink erythema; 2: defined swelling and bright pink erythema, both with distinct borders; 3: defined swelling with a raised border (<1 mm) and bright red erythema with a distinct border; 4: pronounced swelling with a raised border (>1 mm) and dark red erythema. (**b**) The concentration of C-reactive protein (CRP), as well as mean ± standard deviation, was assessed in plasma before and after MVA immunization. Individual levels are shown. (**c**) The concentrations of MCP-1, IL-6, and IP-10 were measured in plasma after the first and second MVA injection. Individual concentrations are shown. The individual AUC after the prime (H3-D14PP) and boost (H3-D14PB) were computed and compared using a permutation test. The mean PP and PB AUC, and the p-values after the permutation test to compare them are indicated. The red arrows indicate prime and boost injections. The dotted line indicates the median concentrations for each cytokine at baseline (D-21PP and H0PP). (**d**) Blood leukocyte counts were followed over time. Individual absolute numbers are shown. Leukocyte counts were missing for macaques BB078 and BB231 at D8PB.

Macaques were subcutaneously injected twice with MVA two months apart (Fig. 1). The specific antibody response developed by each animal was reported in a previous publication, and showed higher serum anti-MVA antibody titers after the boost than the prime^18^. We investigated in the very same animals the early responses to MVA injections. We tested if innate responses differed after prime and boost, as did adaptive immune responses. MVA injections induced low-grade, transient local inflammation at the injection site at early timepoints (Fig. 2a). Inflammation following prime was graded as 1.10 ± 0.22 and resolved by day 3 post-prime (D3PP), whereas it was milder (0.40 ± 0.55) and shorter after the boost.

The blood concentration of C-reactive protein (CRP) showed a transient peak at D1 post-injections (Fig. 2b). This peak was smaller after the boost than the prime (Supplementary Table S1). Among 24 tested soluble factors, only MCP-1, IL-6, and IP-10, were affected by vaccination, with concentrations differing significantly from pre-vaccination levels at two timepoints at least (Fig. 2c, Supplementary Figure S1 and Table S1). MCP-1 and IL-6 were significantly induced after each MVA injection, with an earlier peak post-boost. We also detected high levels of IP-10 early after both the prime and boost. The area under the curve (AUC), as an approximation of exposure over time, showed that the cumulated concentration of IP-10 differed more between each immunization, than those of MCP-1 and IL-6. In addition, although non-significantly impacted, IFNγ, IL-10, IL-13, IL-15, IL-1Rα, IL-5, TGFα and TNFα concentrations tended to follow similar dynamics as MCP-1 and IP-10, whereas IL-1β tended to be closer to IL-6 (Figure S1).

### Blood leukocytes following MVA injection

Changes in local and systemic inflammation after MVA prime-boost immunization were also accompanied by changes in blood cell concentration and composition. The absolute number of leukocytes increased significantly, as early as H3PP and H3PB, and rapidly returned to baseline levels (Fig. 2d and Supplementary Table S2). However, in contrast to the macroscopic local reaction (Fig. 2a), leukocytes AUC were similar after each vaccine injection. Note that these transient post-injections increases of blood cell counts were specific to vaccine injections. Indeed, buffer injection only moderately impacted leukocytes counts, and at a statistically significant lower level at H6 and D1 as compared to MVA (Supplementary Figure S2).

**Figure 3:**
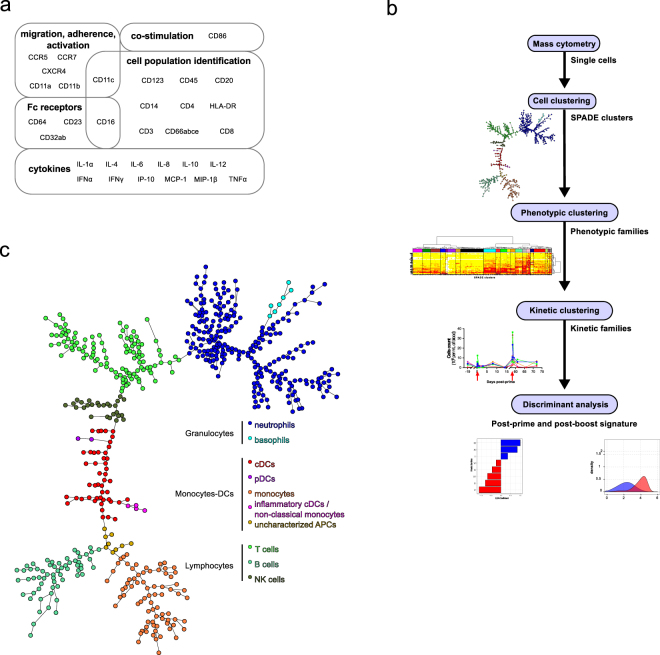
Mass cytometry. (**a**) Fixed leukocytes were stained with a panel of Abs designed to analyze innate myeloid cells by mass cytometry. Thirty-two targeted markers and their associated biological functions are indicated. (**b**) The steps of the mass cytometry data analysis are displayed. As the first analysis step, single cells from FCS files were grouped into clusters sharing similar phenotype using the SPADE algorithm. Clusters were annotated on the resulting SPADE tree based on the expression of a set of 10 markers, and granulocytes and monocytes-DCs were identified. As the second analysis step, clusters of granulocytes and monocytes-DCs sharing the same categories of marker expression were regrouped into phenotypic families. As the third analysis step, phenotypic families sharing the same abundance profiles were clustered into kinetic families. Finally, after these three successive clusterings, and as the last analysis step, discriminant analyses were used to determine which kinetic families best distinguished between post-prime and post-boost immune response, and to define the phenotypic signature of each response. (**c**) Mass cytometry data were analyzed using SPADE. The topology of the SPADE tree is shown. This tree was built using all samples (all macaques and all timepoints). Only the topology of the tree is displayed where each node corresponds to a cell cluster. It does not correspond to a particular sample and size of node is not related to their cell content. Clusters with similar phenotypes are linked using a minimal spanning tree approach. SPADE clusters were annotated and colored with respect to the expression of markers indicated in Supplementary Figure S3 and as follows: neutrophils (CD66^+^), basophils (CD66^-^CD123^+^HLA-DR^−^), monocytes (CD14^+^HLA-DR^+^), cDCs (CD14^−^HLA-DR^+^CD11c^+^CD16^+^), inflammatory cDCs/non-classical monocytes (CD14^+^HLA-DR^+^CD11c^+^CD16^+^), pDCs (CD123^+^HLA-DR^+^), uncharacterized APCs (CD3^−^CD8^−^CD14^−^CD20^−^CD11c^−^CD16^−^CD123^−^HLA-DR^+^), B cells (CD20^+^HLA-DR^+^), T cells (CD3^+^), and NK cells (CD3^−^CD8^+^). Granulocytes were defined as neutrophils and basophils, and monocytes-DCs as monocytes, cDCs, pDCs, inflammatory cDCs/non-classical monocytes and uncharacterized APCs.

Leukocytes correspond to the sum of several immune cell types with heterogeneous phenotypes and functions. Thus, we used mass cytometry to more deeply phenotype blood immune cells over time after immunization, focusing on innate myeloid cells (Fig. 3a and Supplementary Table S3). We developped an analysis pipeline adapt to longitudinal mass cytometry data. We used three successive complementary clusterings, which led to the identification of cell clusters, phenotypic families and kinetic families respectively. They were followed by the automated identification and ranking of the cell populations and markers that best discriminate the responses after the first and the second immunizations (Fig. 3b).

### Mapping the phenotypic diversity of blood granulocytes, monocytes, and DCs

We first used the SPADE algorithm^19^ to identify clusters of leukocytes with similar phenotypes within the whole dataset (all animals and all timepoints). SPADE was optimally parameterized and resulted in the partition of leukocytes into 600 clusters (Supplementary Figure S3 and Tables S5-S6). The stringency of our analysis likely resulted in ‘over-clustering’, generating many artificial cell subpopulations. However, we and others consider ‘over-clustering’ to be less misleading than ‘under-clustering’, particularly as an initial step^21^.

We annotated each cluster based on the SPADE tree and the expression of a restricted set of markers (Fig. 3c and Supplementary Figure S4). We identified 252 clusters of neutrophils (CD66^+^), seven of basophils (CD66^−^HLA-DR^−^CD123^+^), 76 of monocytes (HLA-DR^+^CD14^+^), 47 of CD11c^+^ cDCs latter referred as cDCs (HLA-DR^+^CD14^−^CD11c^+^), four of inflammatory cDCs/non-classical monocytes (HLA-DR^+^CD14^+^CD11c^+^CD16^+^), two of plasmacytoid dendritic cells (pDCs, HLA-DR^+^CD123^+^), and nine of uncharacterized antigen-presenting cells (APCs, HLA-DR^+^CD123^−^CD14^−^CD11c^−^CD16^−^). We focused our analysis on these innate myeloid cells. We also identified 61 clusters of B cells (HLA-DR^+^CD20^+^), 115 of T cells (CD3^+^), and 27 of NK cells (CD3^−^CD8^+^), which were not further studied.

Note that we previously reported that cynomolgus macaque cDCs expressed CD16 at a high and homogeneous level in cynomolgus macaques^10^. The use of CD11c to define cDCs in macaque is controversial. In rhesus and cynomolgus macaques, it has been shown that CD14^high^CD16^high^ and CD14^low/mid^CD16^high^ monocytes expressed higher levels of CD11c than cDCs^22,23^. In contrast, cynomolgus macaques cDCs were recently defined based on their CD11c expression, although it did not allow to fully segregate cDCc into cDC1s and cDC2s by itself and other markers were required^24^. We annotated clusters that shared common features with monocytes and cDCs (HLA-DR^+^CD14^+^CD11c^+^CD16^+^) as inflammatory cDCs/non-classical monocytes, given that there is no consensus yet for cynomolgus macaques DCs/monocytes subsets and that we lacked the critical markers to fully discriminate cDC1s and cDC2s.

We analyzed the dynamics of granulocytes (comprising neutrophils and basophils) (Supplementary Figure S5a and Supplementary Table S2) and monocytes-DCs (comprising monocytes, cDCs, pDCs, inflammatory cDCs/non-classical monocytes and uncharacterized APCs) (Supplementary Figure S5b and Supplementary Table S2) in response to each vaccine injection. We calculated the absolute number of each cell population instead of using the percentage of parent cells because leukocyte counts highly varied during vaccination. Granulocytes represented the most abundant population among leukocytes at all timepoints and showed two significant rapid, transient increases, peaking at H6PP and H6PB. Granulocyte counts returned to baseline levels faster after the boost than the prime. However, their AUC were similar after each vaccine injection. The absolute number of monocytes-DCs also showed a first peak at H6-D1PP and a second, smaller one at H6PB, but without significant differences between the two immunizations.

The local macroscopic reaction likely reflected the recruitment of cells from the circulation and bone marrow to the vaccine injection site^25^. We next deeply characterized the phenotype of granulocytes and monocytes-DCs to reconcile the difference in local and systemic inflammation between prime and boost with the absence of major differences in blood cell counts on the scale of leukocytes, granulocytes, and monocytes-DCs.

**Figure 4:**
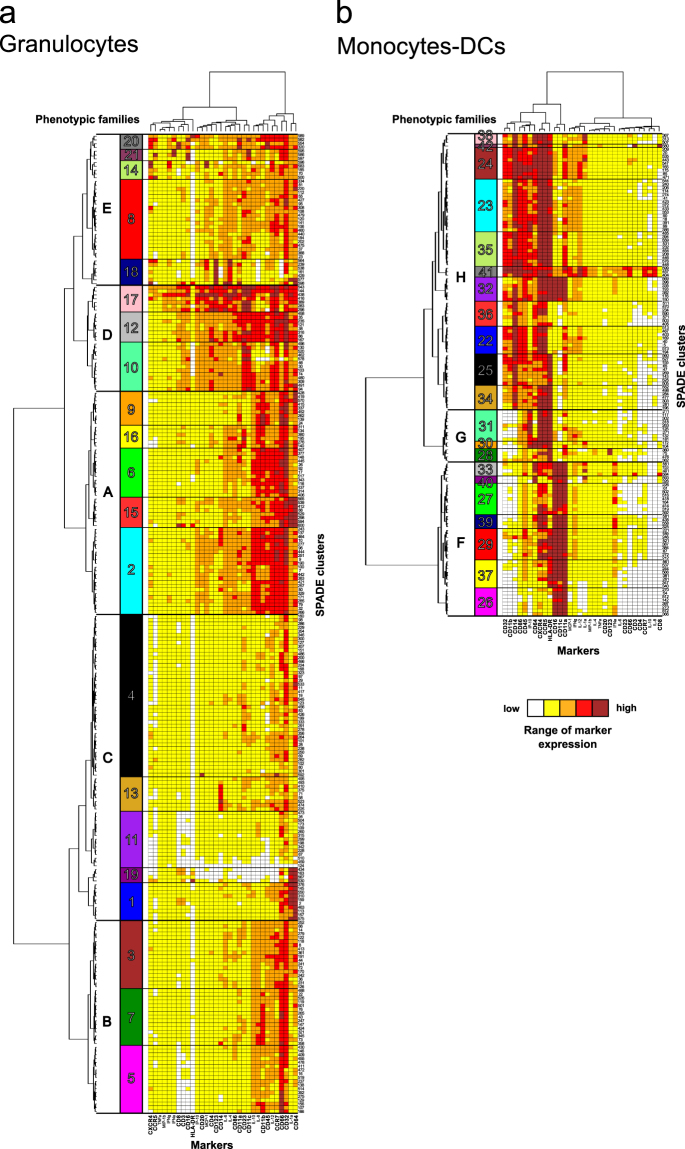
High phenotypic diversity of granulocytes and monocytes-DCs. Hierarchical clustering of markers and (**a**) granulocytes or (**b**) monocytes-DCs clusters were computed and represented as heatmaps. Each line of the heatmaps corresponds to one cell cluster and each column to one marker. Marker and cluster dendrograms were generated to bundle clusters with similar phenotypes and markers with similar expression patterns. Based on the cluster dendrograms, 21 groups of clusters, called phenotypic families, which are arbitrarily colored and numbered using Arabic numerals, were identified for both granulocytes and monocytes-DCs. Groups of phenotypic families defining superfamilies were framed with bold lines, and labeled with capital letters. When describing the heatmaps, phenotypic families are always listed from top to bottom, with ‘;’ to separate them according to their superfamily origin. SPADE clustering markers are written in bold. Interactive heatmaps are available at http://data.idmitcenter.fr/MVA-innate-myeloid/.

SPADE clustering was followed by a second clustering (Fig. 3b). Hierarchical clustering was performed at both cell cluster and marker levels to better visualize the similarities between cell cluster phenotypes and marker co-expression patterns. We generated two categorical heatmaps to visualize phenotype of each cell cluster at a glance, as oppposed to SPADE trees: one heatmap for the granulocytes compartment and one for the monocytes-DCs compartment (Fig. 4). Heatmaps represent the phenotypic diversity within the dataset, but not a particular sample and, in no case, a particular timepoint. They are not snapshots.

Clusters sharing similar phenotypes as measured by a close proximity on the heatmap cluster dendrogram, were gathered into phenotypic families. This analytical strategy prevents inaccurate interpretations due to potential ‘over-clustering’. Indeed, clusters may actually account for different stages of activation or maturation within a cell subpopulation, whereas phenotypic families may represent actual subpopulations.

For the granulocytes compartment (Fig. 4a and http://data.idmitcenter.fr/MVA-innate-myeloid/ for interactive heatmaps), 21 phenotypic families were distributed across five superfamilies, highlighting the richness of their phenotypes. The first superfamily A represented highly activated neutrophils (CD66^mid/high^CCR7^high^CD32^high^CD45^high^CD11b^high^) and comprised phenotypic families 9, 16, 6, 15, and 2. The second superfamily B represented intermediately activated neutrophils (CD66^mid/high^CCR7^mid^CD32^mid^CD45^mid^CD11b^mid^) and contained phenotypic families 3, 7, and 5. The third superfamily C represented poorly activated neutrophils (CD66^low/mid^CCR7^low^CD32^low^CD45^low^CD11b^low^) and comprised phenotypic families 4, 13, 11, 19, and 1. Strikingly, phenotypic family 13 and a subpart of family 2 showed high expression of CD14. CD14^high^ neutrophils were described in the literature^26^ but their functional importance remains to be characterized.

The fourth superfamily D corresponded to CD4^mid/high^CD23^high^CD11c^high^ neutrophils and comprised phenotypic families 17, 12, and 10. The expression of CD4 and CD23 correlated among granulocytes (R = 0.78) (Supplementary Figure S6a). CD4 expression was previously reported in neutrophils^27^ but was not functionally characterized. In addition, the expression of CD23 and CD11c by neutrophils was shown to be linked to inflammation^28–30^. Family 17 showed a high level of several markers, including CD123, CD14, CD86, CD16, IP-10, MCP-1, CD4, CD3, CD8, IL-4, and IL-6. This phenotypic signature may be due to nonspecific staining or to the presence of contaminating cell doublets. It may account for the unexpected strong correlation between the expression of CD3 and CD8 (R > 0.75). One cluster (cluster 416) was annotated as basophils, based on the SPADE tree. Indeed, it showed far higher CD123 expression than its neutrophil counterparts (Supplementary Figure S3), confirming the relevance of our SPADE tree annotation.

The fifth superfamily E included basophils and neutrophils and comprised phenotypic families 20, 21, 14, 8, and 18. Family 18 contained six clusters of CD66^low^CD123^high^IL-4^high^ basophils and one cluster (cluster 598) annotated as neutrophils, based on the SPADE tree. Although high, its CD123 expression was lower than in basophils clusters (Supplementary Figure S3), confirming the relevance of our SPADE tree annotation. Families 20, 21, and 14 corresponded to neutrophils, displaying a unique phenotype characterized by varying expression of CD16, CD11c, CD45, CCR5, CXCR4, MCP1, IFNα, and IFNγ. CD16 and CD11c expression correlated with each other in granulocytes (R = 0.77) (Supplementary Figure S6a), suggesting that phenotypic families 20 and 21 could be highly activated neutrophils and linked to inflammation or modulation of the immune response, with potential IFNα production^31,32^.

We identified four phenotypic families (17, 15, 19, and 1) scattered throughout distinct superfamilies, as well as two clusters of basophils (cluster 577 and 598), expressing a high level of CD64 and IL-1α. The presence of these two markers highly correlated (R = 0.82) with each other (Supplementary Figure S6a). The expression of CD64 in neutrophils was described as a biomarker of infection^33,34^, whereas IL-1α was shown to be important for the recruitment of neutrophils and other myeloid cells^35,36^. In addition, there was a high correlation between IL-12 and CCR7 expression among granulocytes (R = 0.75) (Supplementary Figure S6a). This association suggests a more mature and activated phenotype, since CCR7 was shown to be involved in granulocyte activation^37^ and IL-12 is also key to neutrophil activation^38^.

In the monocytes-DCs compartment, 21 phenotypic families were also identified (Fig. 4b and http://data.idmitcenter.fr/MVA-innate-myeloid/ for interactive heatmaps). These phenotypic families segregated into three superfamilies. The first superfamily F corresponded to cDCs (phenotypic families 33, 40, 27, 39, 29, 37 and 26). The second superfamily G comprised pDCs, CD14^low^monocytes, and uncharacterized APCs (phenotypic families 31, 30 and 28). Finally, the third superfamily H was composed of monocytes and inflammatory cDCs/non-classical monocytes (phenotypic families 38, 42, 24, 23, 35, 41, 32, 36, 22, 25 and 34).

As expected, cDCs and monocytes clearly segregated apart from each other within distinct superfamilies, the former expressing a high level of CD16, CD11c and CD11a, whereas the latter expressed a high level of CD14. CD14^+^CD16^+^HLA-DR^+^ inflammatory cDCs/non-classical monocytes (phenotypic family 32) segregated with monocytes.

CD11a, CD11c, and CD16 expression highly correlated with each other (R > 0.75 for each correlation) (Supplementary Figure S6b), confirming this characteristic of macaque cDCs. cDCs showed varying expression patterns for CCR5, CXCR4, CD64 and HLA-DR. Phenotypic families 29 and 37 consisted of highly activated HLA-DR^high^CCR5^mid/high^CXCR4^mid/high^ cDCs. Phenotypic families 33, 40, 27, 39 and 26 regrouped less activated (potentially immunosuppressive) HLA-DR^low/mid^ cDCs. Phenotypic families 33, 40, 27 and 39 were CCR5^high^CXCR4^high^, whereas family 26 was CCR5^low^CXCR4^low^ suggesting different recruitment abilities to the site of inflammation. In addition, phenotypic families 27 and 39 were CD64^low^ whereas phenotypic families 33 and 40 were CD64^high^ indicating different antibodies binding capacities and suggesting they could be monocytes-derived cDCs^39^.

Besides, CD14, CD11b, and CD32 expression correlated with each other (R > 0.75 for each correlation) (Supplementary Figure S6b), confirming these monocyte-specific features. Surprisingly, CD86 also correlated with CD14 (R = 0.81) and CD32 (R = 0.82) (Supplementary Figure S6b), suggesting that this costimulatory molecule is poorly expressed on macaque blood cDCs, in contrast to monocytes. Overall, monocytes displayed varying expression of CD32, CD11b, and CD45. Phenotypic families 36 and 22 regrouped likely immature and potentially immunosuppressive HLA-DR^low^ monocytes. Phenotypic families 25 and 34 regrouped poorly activated CD32^low^CD11b^low^HLA-DR^high^ monocytes. Phenotypic family 32 consisted solely of inflammatory cDCs/non-classical monocytes. Phenotypic families 23 and 35 consisted of moderately to highly activated CD32^mid/high^CD11b^mid/high^CD11a^mid^HLA-DR^high^ monocytes. Finally, phenotypic families 38, 42 and 24 consisted of highly activated CD32^high^CD11b^high^CD11a^high^HLA-DR^high^ monocytes. Phenotypic family 41 consisted of IL-12 producing CD66^high^CCR7^high^HLA-DR^high^ monocytes.

In the second superfamily G, clusters expressed a high level of HLA-DR and segregated closer to monocytes than cDCs. Phenotypic families 31 and 30, labeled as uncharacterized APCs, contained CCR5^high^CXCR4^high^IP-10^high^ cells. These cells could correspond to activated monocytes which have downregulated CD14 and CD11b, a feature linked to activation and macrophage differentiation^40,41^. Family 28 contained two CD123^high^IL-4^mid^ clusters, labeled as pDCs (clusters 360 and 75), and two clusters labeled as monocytes, that were solely HLA-DR^high^CD14^low^ (clusters 476 and 362). Interestingly, the two clusters of pDCs differed by the expression of CCR5 and CXCR4, suggesting different recruitment and/or maturation abilities. Most pDCs were CCR5^high^CXCR4^high^ but surprisingly IFNα^low^ (Supplementary Figure S3). This may be explained by the *ex vivo* staining without cytokine secretion inhibitors. Indeed, whole blood was extemporaneously fixed after sampling and without blocking cytokine secretion to avoid granulocytes alteration. Using these settings, we may not capture the production of cytokines whose concentrations did not reach a certain threshold or that were secreted very fastly. Alternatively, blood pDCs may be non-activated and thus would not produce IFNα^42^. In addition, we may not capture all the pDC diversity due to the low amount retrieved from macaque blood (Supplementary Figure S7).

Our data show a high degree of phenotypic diversity among blood granulocytes and monocytes-DCs with several degrees of activation/maturation. We uncovered potential novel subsets with newly described marker co-expression. Such diversity was captured because of the high number of markers specifically targeting innate myeloid cells and the high number of clusters defined. However, this diversity was also captured because our original longitudinal dataset was composed of samples collected at steady state, before or long after immunization, outnumbered by samples collected early after immunization, during acute inflammation.

### Distinct subphenotypes respond to first and second immunizations

**Figure 5:**
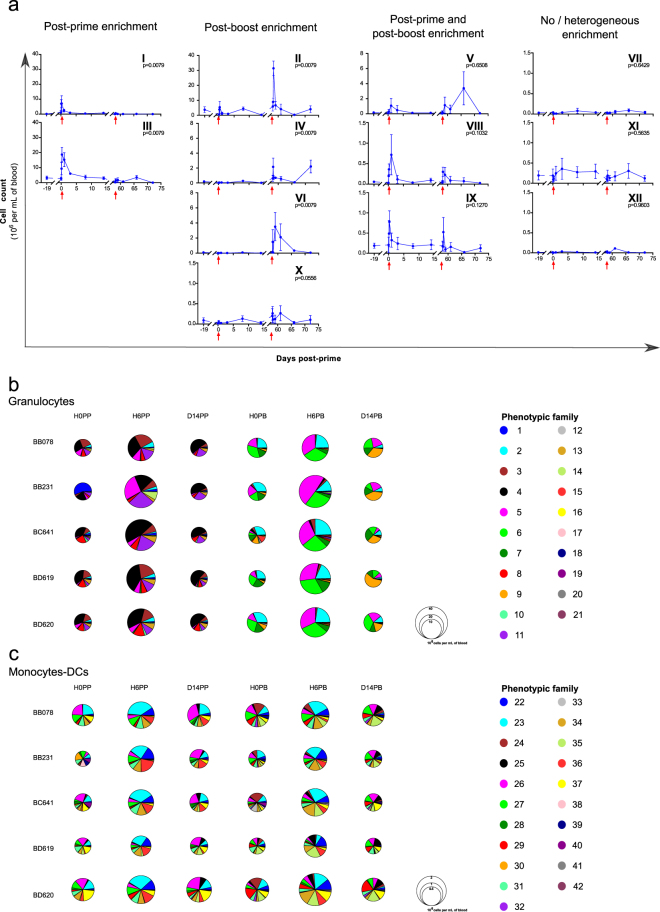
Different enrichment of innate myeloid cells after first and second immunizations. (**a**) Phenotypic families sharing similar abundance profiles were gathered into kinetic families after hierarchical clustering based on abundance profiles with the Pearson correlation. Twelve kinetic families were defined and arbitrarily numbered from I to XII. They were further regrouped based on their kinetic pattern with an enrichment essentially post-prime, essentially post-boost, both after the prime and boost, or no or heterogeneous enrichment after each immunization. The mean abundance among the five animals is displayed ± standard deviation. The individual AUC after the prime (H3-D14PP) and boost (H3-D14PB) were calculated for each kinetic family and compared using a permutation test. The p-values are indicated and considered to be significant when p ≤ 0.01. Note that the scale of the Y-axis is specific to each kinetic family. The red arrows indicate the prime and boost injections. (**b**,**c**) Composition in phenotypic families of the granulocytes (**b**) and monocytes-DCs (**c**) compartment at H0PP, H6PP, D14PP, H0PB, H6PB and D14PB for each macaque. The size of the pie chart is proportional to the cell concentration. The color-code for each phenotypic families is identical for the pie-charts and the heatmaps.

The categorical heatmaps that we generated provided good information on cell subsets phenotypic diversity within the dataset. But these still pictures did not provide information on cell subset frequencies, or their continuous or transient presence. To go beyond these pictures, we studied the impact of MVA injections on the abundance of cells from the different phenotypic families with respect to animals and time. Various abundance profiles depending on the phenotypic families were found and the post-prime and post-boost transient expansions were not always equal (Supplementary Figures S8 and S9). Using a third clustering step (Fig. 3b), the 21 phenotypic families of granulocytes and 21 phenotypic families of monocytes-DCs were gathered together into 12 kinetic families sharing similar abundance profiles (Fig. 5, Supplementary Figures S9, S10 and Supplementary Table S4). The correspondence between kinetic families and phenotypic families is given in Table 1 and in Supplementary Figures S9 and S10, but more simply thanks to interactive heatmaps on http://data.idmitcenter.fr/MVA-innate-myeloid/.

**Table 1:** Correspondence between kinetic and phenotypic families.

Kinetic family	Composition		Kinetic pattern
	Phenotypic family	Cell population	
I	Granulocytes 14; 11, 19, and 1 Monocytes-DCs 30	Neutrophils, including poorly to moderately activated neutrophils and uncharacterized APCs	Post-prime enrichment
III	Granulocytes 8; 4 and 13; 3		
II	Granulocytes 6 and 2; 7 and 5 Monocytes-DCs 35 and 41	Neutrophils, including moderately to highly activated neutrophils, highly activated monocytes, CCR5^high^CXCR4^high^cDCs, and inflammatory cDCs/non-classical monocytes	Post-boost enrichment
IV	Granulocytes 9 and 15		
VI	Granulocytes 12 and 16		
X	Monocytes-DCs 24 and 32; 39 and 29		
V	Granulocytes 20; 17 and 10 Monocytes-DC 42 and 25	Neutrophils, poorly to highly activated monocytes, HLA-DR^low^cDCs, and uncharacterized APCs	Post-prime and post-boost enrichment
VIII	Granulocytes 21 Monocytes-DCs 38, 36 and 22		
IX	Monocytes-DCs 23 and 34; 31; 27		
VII	Granulocytes 18	Basophils, pDCs, CD14^low^monocytes, and cDCs including HLA-DR^low^and CD64^high^cDCs	No/heterogeneous enrichment
XI	Monocytes-DCs 28; 37 and 26		
XII	Monocytes-DCs 33 and 40		

For each kinetic family, its composition in terms of phenotypic families (listed from top to bottom from the corresponding heatmaps (Fig. 4) and separated by “;” to designate their being from different superfamilies) and its main cell populations and phenotypes, as well as its kinetic pattern, as classified in Fig. 5a, are indicated.

Strikingly, although there was no significant difference between prime and boost leukocytes AUC (Fig. 2d), nor granulocyte AUC or monocytes-DCs AUC (Supplementary Figure S5), the dynamics of five of the 12 kinetic families displayed significant differences (measured with AUC) after the prime and boost. Poorly to moderately activated neutrophils (granulocytes phenotypic families 14, 8, 4, 13, 11, 19, 1, 3), as well as uncharacterized APCs (monocytes-DCs phenotypic family 30) belonged to kinetic families I and III, and were mostly present in the blood after the prime (Fig. 5a and Supplementary Figure S10). The enrichment of cells from kinetic family III was not as transient as those of cells from kinetic family I.

Conversely, moderately to highly activated neutrophils (granulocytes phenotypic families 12, 9, 16, 6, 15, 2, 7, and 5), activated monocytes (monocytes-DCs phenotypic family 24, 35, and 41), inflammatory cDCs/non-classical monocytes (monocytes-DCs phenotypic family 32), and CCR5^high^CXCR4^high^cDCs (monocytes-DCs phenotypic family 39 and 29) composed kinetic families II, IV, VI and X, and were mostly present after the boost (Fig. 5a and Supplementary Figure S10). We observed two successive waves of enrichment for kinetic families IV, VI, and X. The second wave was especially late for family IV.

Neutrophils (granulocytes phenotypic families 20, 21, 17, and 10), monocytes (monocytes-DCs phenotypic families 38, 42, 23, 36, 22, 25, and 34), uncharacterized APCs (monocytes-DCs phenotypic family 31), and HLA-DR^low^cDC (monocytes-DCs phenotypic family 27) were part of kinetic families V, VIII, and IX, and were both affected after the prime and boost, albeit differently (Fig. 5a and Supplementary Figure S10). For kinetic family V, there was only one rapid and transient increase at D1PP, whereas there were two rapid and transient increases at D1 and D8PB, the second being stronger. Kinetic family VIII showed an increase at H6-D1 post-injections with a stronger and faster increase after the prime. Kinetic family IX displayed a rapid and transient increase at H6 post-injections, the PP peak being larger.

There was no significant impact of MVA immunizations on cell abundance for the basophils (granulocyte phenotypic family 18), pDCs, CD14^low^ monocytes (monocytes-DCs phenotypic family 28), as well as HLA-DR^low^ and CD64^high^ cDCs (monocytes-DCs phenotypic family 33, 40, 37 and 26) belonging to the three remaining kinetic families VII, XI, and XII (Fig. 5a and Supplementary Figure S10). Cells from these kinetic families were scarce.

To provide a more general picture of the distribution of the different phenotypic families and its evolution, pie charts were displayed at key timepoints: just before the prime (H0PP), just before the boost (H0PB), at the acute peak of the innate immune response (H6PP and H6PB), and at later timepoints (D14PP and D14PB) (Fig. 5b,c). This representation highlighted that, strikingly, the composition in phenotypic families already differed just before the boost as compared to just before the prime. Also, at D14PP, granulocyte counts were back to baseline, but the repartition in phenotypic families was still closer to H0PP than H0PB, suggesting that innate myeloid responses were not over at D14 post-injection and that a switch in phenotype occurred later, between D14PP and D58PP/H0PB. Differences between prime and boost were not as pronounced within the monocytes-DCs compartment.

### Biological relevance of kinetic families

Before going further, we addressed the relevance of the kinetic families identified through three successive clustering steps (Fig. 3b). We assessed whether there were any associations between a direct measurement of inflammation and the enrichment of these kinetic families.

**Figure 6:**
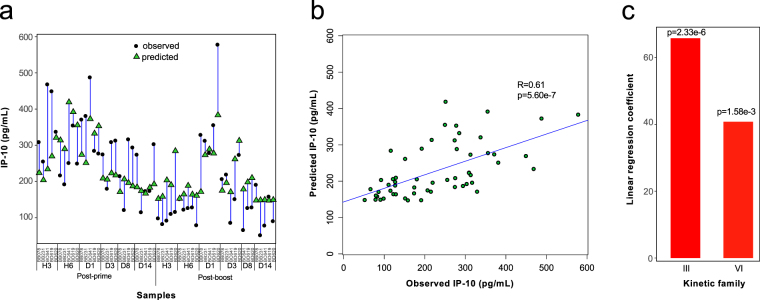
Association of kinetic families with a direct biological measurement. (**a**) The relationship between kinetic family abundances and IP-10 plasma concentration was analyzed using linear regression. Iterative linear regressions were generated until all coefficients had a p-value ≤0.05. At each iteration, the coefficient having the highest p-value higher than 0.05 was removed. The differences between predicted *vs*. observed concentrations of IP-10 are shown. (**b**) The ELISA measured and linear regression predicted IP-10 concentrations are shown in a bi-plot representation. The Pearson correlation coefficient and p-value are indicated. (**c**) The linear regression coefficients of kinetic families III and VI, which are necessary and sufficient to predict IP-10 concentrations, are displayed along with their p-values.

We used a linear regression analysis to predict IP-10 concentration based on kinetic family abundances. We chose IP-10 because it differed the most in AUC between prime and boost. IP-10 was also proposed as a candidate biomarker for diagnosis, prognosis, or responsiveness to therapy for several inflammatory and infectious diseases^43^. The prediction highly fit our observations (Fig. 6a,b), validating the computational analysis designed to define kinetic families. Linear regression showed that IP-10 concentrations positively correlated with the abundance of two kinetic families, III and VI (Fig. 6c), corresponding to neutrophils enriched only after the prime (granulocyte phenotypic families 8; 4 and 13; and 3) and only after the boost (granulocyte phenotypic families 16 and 2) (Table 1). These neutrophils surprisingly did not produce IP-10, in contrast to granulocyte phenotypic family 17. It is possible that plasma IP-10 was mainly released by cells present at the MVA injection site rather than in blood.

### Key phenotypic features that discriminate between post-prime and post-boost immune responses

Our findings show that innate myeloid immune responses strongly differed between prime and boost, but it did not take into account the absolute number of cells in each kinetic family. Thus, we performed a Multidimensional Scaling (MDS) analysis to represent the similarities between all samples, based on cell abundances of each kinetic family.

**Figure 7:**
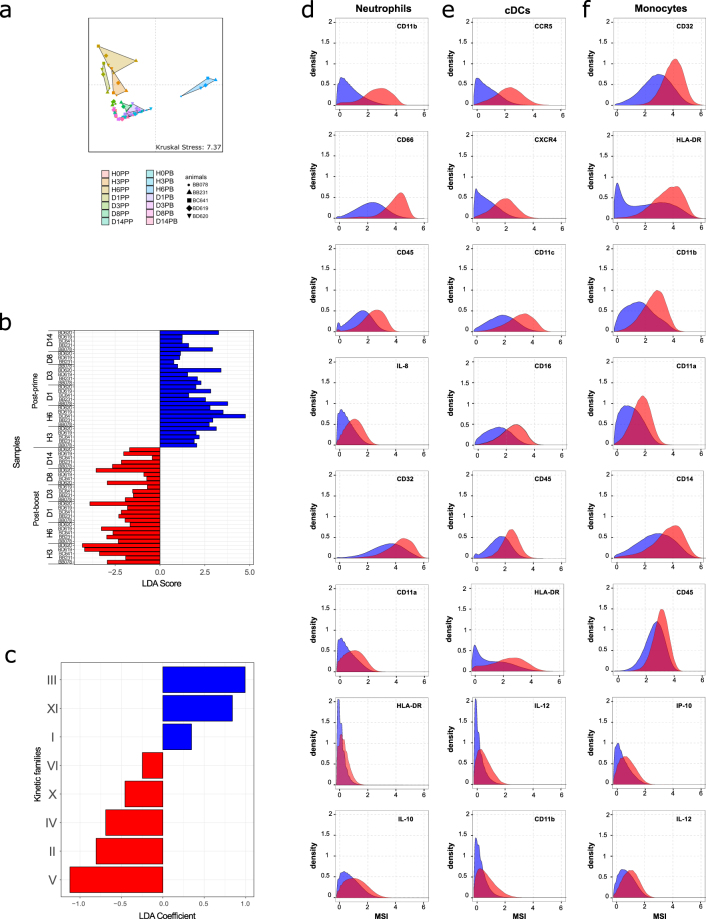
Visualization of and discrimination between post-prime and post-boost innate myeloid cell responses. (**a**) The Multidimensional Scaling (MDS) representation was calculated based on the abundance of each kinetic family. The Kruskal Stress is indicated and corresponds to the percentage of information lost during the dimensionality reduction process. Samples collected long before the prime injection (D-19PP) were not included. (**b**) Linear Discriminant Analysis (LDA) was performed after Least Absolute Shrinkage and Selection Operator (LASSO) (Supplementary Figure S11). Samples from the timepoints D-19PP, H0PP, and H0PB were not used for this analysis. The LDA scores of each sample are shown. The LDA score indicated whether a given sample was classified as post-prime (positive score) or post-boost (negative score). The samples colored in blue correspond to post-prime samples and the samples colored in red correspond to post-boost samples. (**c**) The LDA coefficients for each kinetic family are shown. (**d–f**) Kolmogorov-Smirnov (KS) distances of expression distribution densities were computed for each marker for neutrophils, cDCs, and monocytes (Supplementary Figure S12). This distance corresponds to the maximal difference between the distributions of marker expression in the two compared cell populations. KS distance is commonly used in flow cytometry analyses^93^. MSI histograms for the top 8 markers with the highest KS distance are displayed for (**d**) neutrophils, (**e**) cDCs, and (**f**) monocytes from kinetic families that discriminate between the post-prime (blue) and the post-boost responses (red) as defined in Fig. 7c. Histograms were built on the whole dataset and did not represent a particular sample (animal or timepoint).

H3PP, H6PP, D1PP, and H6PB clearly segregated apart from most timepoints, which were close to one another in the MDS representation (Fig. 7a). H3PP, H6PP and D1PP were also distant from H6PB. This segregation confirmed that innate myeloid responses differed between prime and boost. It also showed that the innate immune response was strongly affected during three timepoints post-prime and only a single timepoint post-boost.

We further identified the main features that differed between the post-prime and post-boost immune response by using a Least Absolute Shrinkage and Selection Operator (LASSO) approach (Supplementary Figure S11). This approach allowed to statistically select the kinetic families that best characterized the post-prime or post-boost immune response. In particular, it permitted to exclude, in a statistical manner, kinetic families that were not impacted by vaccination or were impacted similarly at both immunizations. Among the twelve kinetic families, eight (I, II, III, IV, V, VI, X and XI) were sufficient to fully discriminate between the two responses (Fig. 7b). Linear discriminant analysis (LDA) allowed us to score their contribution to post-prime and post-boost category and gave a statistical criterion to classify the selected kinetic families in post-prime or post-boost signature (Fig. 7c). This analysis revealed that the main components of the post-prime immune response were kinetic families III, XI, and, to a lesser extent, I. They were composed of granulocyte phenotypic families 14 and 8; 4 and 13; 11, 19, and 1; and 3 (poorly to moderately activated neutrophils) and monocytes-DCs phenotypic families 30 and 28; and 37 and 26 (pDCs, CD14^low^ monocytes, uncharacterized APCs, and cDCs). Conversely, the main components of the post-boost immune response were kinetic families V, II, IV, X, and, to a lesser extent, VI. They were composed of granulocyte phenotypic families 20; 17, 12, and 10; 9, 16, 6, 15, and 2; and 7 and 5 (moderately to highly activated neutrophils) and monocytes-DCs phenotypic families 42, 24, 35, 41, 32 and 25; and 39 and 29 (monocytes, including inflammatory cDCs/non-classical monocytes, and cDCs) (Table 1).

We identified markers differentially expressed by cell populations that best discriminated the post-prime from the post-boost response, based on a Kolmogorov-Smirnov distance criterion (Supplementary Figure S12). The expression of CD11b, CD66, CD45, IL-8, CD32, CD11a, HLA-DR and IL-10, and to a lesser extent CCR7, was higher in neutrophils found in abundance in blood after boost than after prime (Fig. 7d and Supplementary Figure S12a). CCR5, CXCR4, CD11c, CD16, CD45, IL-12, HLA-DR, and CD11b were more highly expressed by cDCs enriched after the boost than those enriched after the prime (Fig. 7e and Supplementary Figure S12b). To a lesser extent, CD11a expression was also associated with cDCs enriched after the boost. Finally, CD32, CD11b, HLA-DR, CD11a, CD14, CD45, IP-10, and IL-12 expression was higher in monocytes involved in the post-boost response than those participating in the post-prime response (Fig. 7f and Supplementary Figure S12c).

In conclusion, innate myeloid subpopulations that distinguished the post-boost from the post-prime response, and that were actually already present at the time of the boost, showed higher expression of several activation/maturation markers. Among them, three markers were shared by neutrophils, cDCs, and monocytes: CD45, CD11b and HLA-DR.

## Discussion

We report a mild local reactogenicity to subcutaneous MVA injection in macaques, which was weaker and more rapid after the second injection. Plasma CRP and IP-10 concentrations, used as systemic inflammation readouts, were also more attenuated after the boost. We observed an early and transient enrichment of innate myeloid cells in the circulation both after the prime and the boost. Strikingly, at the level of each compartment (granulocytes and monocytes-DC), no major differences in cell abundance intensity or kinetics were observed between the two injections. Qualitative differences between the blood innate myeloid cell responses after one or two immunizations were only observable after deep phenotyping using mass cytometry combined with an analysis pipeline consisting of three successive clusterings specifically developed for longitudinal multidimensional data, and a final discriminant analysis. Neutrophils, monocytes and DCs, which expanded transiently at each immunization, were not composed of the same cell subpopulations. While circulating innate myeloid cells rapidly returned to baseline level in terms of their number after prime, their sub-phenotype composition was modified over time, and finally different at the time of the boost. Some subsets expanded after each immunization, whereas others were enriched primarily after the prime or the boost. Neutrophils, monocytes, and DCs responding to the second injection expressed higher levels of CD45, HLA-DR, Fc receptors CD16 and CD32, integrins CD11a, CD11b, and CD11c, some of which form complement receptors CR3 (CD11b/CD18) and CR4 (CD11c/CD18), chemokine receptors CCR5 and CXCR4, pro-inflammatory cytokines IL-8, IL-12, and IP-10, and anti-inflammatory cytokine IL-10. Thus, local and systemic inflammation after the boost was attenuated with respect to after the prime, whereas as many innate myeloid cells were recruited after the boost as after the prime. However cells mobilized after the boost were more activated and mature. We also highlight the importance of neutrophils, in addition to professional APCs monocytes and DCs, in the early response to MVA, unveil their high degree of phenotypic heterogeneity, and discover new subsets.

The transient mobilization of leukocytes was specific to the vaccine, since buffer injection did not result in such a dramatic effect. However, we cannot formally rule out a role played by the recombinant fusion protein HIV gag-pol-nef encoded in our vaccine.

The mild local adverse reaction is typical of subcutaneously administered vaccines and was expected for a non-replicating vaccine such as MVA. The subcutaneous injections of IMVAMUNE® or ACAM3000, which are third generation MVA vaccines against smallpox, have been described as safe, in contrast to replicating VACV which is more reactogenic^44,45^. The local reaction to VACV and MVA was reported to be stronger and longer-lasting for primary vaccinees than for non-naive participants, and after the first injection than after the second one^44,46,47^. This was also supported by our data on MVA vaccination in macaques.

We found that IL-6, MCP-1, and IP-10 plasma concentrations were altered after subcutaneous injections of cynomolgus macaques with MVA. The differences between post-prime and post-boost AUC were higher for IP-10. There are only limited *in vivo* studies on the early plasma cytokine response to MVA, contrary to *in vitro* data after restimulation of PBMCs or *in vivo* studies on earlier generation smallpox vaccines, for which correlations between cytokine expression patterns and adverse events or vaccine take were identified. A study in rhesus macaques showed that IL-6 and IP-10 were the only cytokines for which levels increased in plasma one day after a single MVA intramuscular injection^48^. Comparison of serum cytokines after primary vaccination or re-vaccination with VACV in human volunteers showed that the peak levels were also statistically different for IP-10, in addition to IFNγ and MIG (CXCL9)^49^, which are induced by IFNγ and/or IFN type I and interact with the same receptor, CXCR3^43^. Among many other soluble factors, IL-6, MCP-1, and IP-10 are produced *in vitro* in response to MVA by whole blood, PBMC, primary human monocytes and macrophages^50,51^, and the pathways involved have been elucidated^51,52^. The capacity of MVA to induce MCP-1 production distinguished it from other VACV strains^50^. Whether they are produced by infected cells and/or bystander cells^53^ is yet to be fully determined. Whether their presence in plasma originates from their release by cells from the injection site and/or from blood cells requires further investigation.

Beyond reactogenicity and plasma cytokine responses, the novelty of our study is the analysis of early innate cellular events after vaccination. Innate responses in blood or locally after injection of adjuvants or vaccines, including MVA, have been characterized in mice or macaques to better understand the mode of action of vaccines^54–59^. However, none of these studies analyzed the early cellular response to vaccines in the context of prime/boost strategies. One seminal study^54^ compared distinct TLR adjuvants in rhesus macaques and demonstrated that they differentially stimulated systemic immune responses. The intensity quality and kinetics of blood cells enrichment were specific of each adjuvant. MPL, R848, and CpG ODN induced a rapid increase of blood neutrophils and CD14^+^ monocytes. However, neutrophil counts reached a higher level with R848 and two successive neutrophil expansions were observed with CpG ODN. In addition, only R848 and CpG ODN mobilized the intermediate CD14^+^CD16^+^ followed by non-classical CD14^dim^CD16^high^ monocytes and mediated pDCs and cDCs activation.

We identified potential new subsets of innate myeloid cells. There were more CD4^high^CD23^high^CD11c^high^ neutrophils after the boost than the prime. They are likely linked to inflammation^27–29^, and may exert regulatory/suppressive effects. CD66^high^CD32^high^CD11b^high^CD45^high^CCR7^high^IL-10^high^ neutrophils were also more highly enriched after the boost than the prime^37^. They may have a critical role in the resolution of infection upon the second encounter with pathogens. As previously described^10,60–63^, we observed a population of cells sharing cDC and monocyte phenotypic patterns (CD11c^high^CD16^high^CD11a^high^CD14^high^CD45^high^IP-10^mid/high^), which we designated inflammatory cDCs/non-classical monocytes. This pro-inflammatory cell type expanded more after the boost than the prime. We also identified HLA-DR^low^ monocytes and cDCs, which are likely immature or immunosuppressive. Comparative functional analyses are required for their definitive classification as new subsets. In future studies, a refined panel should also include antibodies targeting additional markers, such as CD1c, CD141, CD172a, CD33, CD45RA and SIGLEC6, to clearly distinguish DC precursors from pDCs, separate the various cDC and monocyte subtypes, and identify myeloid-derived suppressive cells (MDSCs)^24,64–66^.

Innate immune responses differ after one and two immunizations, as adaptive immune responses do. There are many possible reasons for this difference which are yet to be tested. At the boost, there were antibodies directed against MVA, contrary to the prime^18^. The vaccine was probably rapidly cleared and sensed differently, not as free viral particles, but as immune complexes. A different sensing could translate into a different alarm. It was recently shown, using mass cytometry, that the activation of signaling pathways and cytokine production by blood innate myeloid cells in response to the split influenza vaccine was dependent on immune complex formation and CD16 and CD32 FcγR activation^67^. Several studies, based on monoclonal antibody therapies, have also reported the importance of immune complexes and Fc receptor engagement in enhanced protective immune responses and vaccine-like effect^7^. This would be consistent with the higher expression of the FcR (CD16 and CD32) we observed among blood neutrophils, monocytes, and cDCs responding to the boost. Local memory cells established after the first immunization could also have played a role in the different innate myeloid responses after one and two immunizations. It has been shown in mice that IFNγ and MIP-1α produced by memory T cells after antigen recognition resulted in faster activation and recruitment of innate cells as well as better killing capacities by phagocytes^68,69^. In addition, TNFα produced by resident memory CD8^+^ T cells was reported to induce local DC maturation^70^. The induction of resident CD8^+^ T cells in the skin has been demonstrated in mice after MVA^71^ and VACV skin infection^72,73^. Finally, trained innate immunity^5^ could participate in the different innate immune responses during prime/boost immunizations, assuming that trained cells survive long enough between the two immunizations. Tissue-resident macrophages and/or cells recruited from blood or their progenitors after the prime could be involved.

Conversely, the different innate responses after the prime and boost may affect the restimulation of primary memory B and T cells and their differentiation into secondary memory cells. Neutrophils the most specific for the post-boost response expressed higher levels of CD66, which plays a role in adhesion and interactions with DCs^74^, and of IL-8, a key chemoattractant of neutrophils to the site of inflammation and inducer of phagocytosis^75^. Neutrophils expanding after the boost also produced more IL-10. Immunosuppressive IL-10 producing neutrophils have been reported in mice^76,77^, whereas their presence in humans is more controversial^78^. Finally, neutrophils recruited after the boost expressed a higher level of CCR7, suggesting their capacity to migrate directly to lymph node^37,79^. cDCs and monocytes, and to a lesser extent neutrophils, participating in the early response after the boost expressed higher levels of HLA-DR, involved in antigen presentation to CD4^+^ T cells, and IL-12, promoting Th1 development and controlling the CD8^+^ T cell response^80^. Neutrophils, cDCs, and monocytes mobilized after the boost were more prone to phagocytosis with higher expression of FcR (CD32 and CD16) and integrins CD11a, CD11b and CD11c, which are also involved in tissue-specific homing of leukocytes during inflammation and leukocyte activation^81,82^. Monocytes were more activated, as they expressed a higher level of CD14, which acts as PRR^83^, and IP-10. cDCs were also more mature, with a higher expression of CXCR4 and CCR5, which allow their trafficking to the vaccine injection site and its draining lymph node^84,85^. Finally, innate myeloid cells elicited after the boost were more responsive with higher expression of CD45. CD45 is well known to lower the threshold of BCR and TCR signaling on B and T cells. It was also shown to regulate FcR, TLR, and cytokine signaling in phagocytes and DCs, as well as neutrophils migration^86^. Overall, better antigen uptake and presentation is more likely after the boost than after prime, contributing to enhanced T cells restimulation, although the requirements of signal 1 (TCR stimulation), 2 (co-stimulation), and 3 (inflammatory cytokines) differ between naive and memory T cells activation^87^.

Whether the observed differences between the early response to the first and second vaccine injections also hold true for innate lymphoid cells needs to be tested. Additionally, it is important to define what is MVA-specific from what is shared with other vaccines. Another challenge will be to determine predictive correlations between innate and adaptive responses in the context of prime/boost immunizations, as previously done for one-dose vaccines^88^, or yearly influenza vaccine^89^. Nonetheless, the differential innate responses described here can be valuable to tailor vaccine-induced immunity.

## Methods

### Ethics statement

This experiment was approved by the «Ministère de l’Éducation Nationale, de l’Enseignement Supérieur et de la Recherche» (France) and the ethical committee «Comité d'éthique en expérimentation animale n°44» (France) under the reference 201503131451825402 (APAFIS#319) and 2015062215324227v1(APAFIS#891) for Figure S2. Animals were handled by veterinarians in accordance with national regulations (CEA Permit Number A 92–32–02) and the European Directive (2010/63, recommendation N°9) and in compliance with Standards for Human Care and Use of Laboratory of the Office for Laboratory Animal Welfare (OLAW, USA) under OLAW Assurance number #A5826-01.

### Vaccine, animals, and blood samples

The ANRS recombinant MVA HIV B vaccine (MVATG17401; Transgene, Illkirch-Graffenstaden, France) was injected subcutaneously into five cynomolgus macaques, at 4 × 10^8^ PFU, two months apart as previously described^18^. It contains the full-length codon-optimized sequence of *gag* (encoding amino acids [aa] 1 to 512) fused with fragments from *pol* (encoding aa 172 to 219, 325 to 383, and 461 to 519) and *nef* (encoding aa 66 to 147 and 182 to 206) from the Bru/Lai isolate (Los Alamos database accession number K02013). Blood samples were collected longitudinally in EDTA, to count leukocytes, or Lithium-Heparin to measure soluble plasma factors and perform single-cell mass cytometry analyses.

For Figure S2, six macaques were injected subcutaneously with a buffer containing 10 mM Tris-HCl, saccharose 5% (w/v), 10 mM NaGlu, 50 mM NaCl, pH8.0 and one month later with the same dose and batch of MVA HIV B as the other five animals.

### Quantification of plasma soluble factors

C-reactive protein (CRP) was quantified in plasma by Laboratoire Vébio (Arcueil, France) using an immunoturbidimetry assay (CRP Plus, Thermo Scientific). Cytokine, chemokine and growth factor levels were assessed with a multiplex immunoassay (MILLIPLEX MAP non-human primate cytokine magnetic bead panel, Millipore), except plasma IP-10 concentrations, which were assessed by ELISA (human CXCR10/IP-10, R&D systems). Post-prime (PP) and post-boost (PB) samples were assessed independently.

### Staining, mass cytometry acquisition, and data processing

Blood processing, staining, and acquisition using a CyTOF (Fluidigm), as well as initial leukocyte gating, were performed as previously described^10^. Briefly, 1 mL of blood was incubated with a fixation mixture containing PFA and glycerol^10,90^ for 10 min at 4 °C. After centrifugation, erythrocytes were lysed in 10 mL of milli-Q water at room temperature for 20 min. Cells were then washed in DPBS 1× and stored at −80 °C at a final concentration of 15 · 10^6^ cells in the fixation mixture. Three millions of fixed leukocytes were thawed. After 2 washes with PBS/BSA at 0.5%, they were incubated with the surface antibodies at 4 °C for 30 min (Supplementary Table S3). They were washed twice in PBS 1× and fixed in PBS/PFA 1.6% for 20 min RT. After permeabilization in Perm/Wash Buffer 1× (BD Biosciences) for 10 min at RT, cells were incubated with intracellular antibodies at 4 °C for 30 min (Supplementary Table S3). Finally cells were washed in PBS and incubated overnight with 0.1 µM of iridium RNA/DNA intercalator in PBS/PFA at 1.6%. The next day, cells were washed three times with milli-Q water and filtered using a 35 µm nylon mesh cell stainer (BD Biosciences). EQ^TM^ four elements calibration beads (Fluidigm, San Fransisco, USA) were added following manufacturer’s protocol. Each sample was divided into two replicates and acquisition was performed using the autosampler device for CyTOF (both from DVS Fluidigm). 5 stainings/acquisitions were done (one per animal) using the same batch of antibodies each time. In addition, we followed an established strategy^91^ to control the quality of each staining/acquisition and their reproducibility by including the same two control samples (Supplementary Figure S13).

### Automatic identification of cell populations

Cell populations were identified using the Spanning-tree Progression Analysis of Density-normalized Events (SPADE) algorithm^19^. Briefly, a random pre-downsampling was used to select 60,000 cells from each sample (60,000 corresponded to the number of cells contained in the smallest sample -Table S5). Then the SPADE algorithm *per se* was applied to all samples (all macaques and all timepoints) to define the phenotype of each cluster as well as the topology of the tree. Full upsampling was eventually performed.

For our dataset, the optimal SPADE settings were determined with SPADEVizR package^20^ as 20 clustering markers (CD66, HLA-DR, CD3, CD64, CD8, CD123, CD11a, CD11b, CD4, CD23, CD86, CD32, CXCR4, CCR5, CD16, CD11c, CD14, CD45, CD20 and CCR7), 600 clusters, a density-based downsampling of 10%, and an outlier density parameter of 0.01. The clustering quality was expressed as the percentage of clusters displaying a unimodal and narrow distribution of all clustering markers, as well as the percentage of small clusters (clusters with less than 50 cells in total). Markers distributions were assessed using the Hartigan’s dip test (p-value < 0.05 to reject the uni-modality hypothesis). Markers distributions with an interquartile range (IQR) < 2 were considered to be narrow. These settings resulted in the highest percentage of uniform clusters and the absence of small clusters. Numbers and percentages of non-uniform clusters for each marker are displayed in Table S6.

### Leukocyte counts, absolute number calculation, and abundance profiles

The leukocyte counts were quantified using an HmX instrument (Beckman Coulter). The absolute number of cells in a population was computed as: N = the absolute number of leukocytes expressed per μL of blood x number of cells in the population detected by the CyTOF/total number of leukocytes (defined as non CD3^+^CD66^+^ cells) detected by the CyTOF. The absolute number kinetics was called the abundance profile.

### Heatmap representations of the cell cluster phenotypes

Heatmaps of the cell cluster phenotypes were generated using SPADEVizR^20^. The mean of the median of the mean signal intensity (MSI) for each marker among samples was displayed according to five phenotypic bins calculated by dividing the marker range of expression between the 5^th^ and the 95^th^ percentile into five categories for all cell clusters. For each cluster, samples contributing less than 10 cells were excluded. Hierarchical clusterings of cell clusters and markers were performed using the Euclidean metric based on the ward.D linkage.

### Phenotypic and kinetic families

Cell clusters sharing similar phenotypes were gathered into phenotypic families based on the cluster dendrogram. Phenotypic families sharing similar dynamics were gathered into kinetic families based on their abundance profiles. This determination was performed using SPADEVizR^20^ with the hierarchical method based on the Pearson correlation and complete linkage.

### Statistical tests

Soluble factor concentrations and cell abundances were compared between timepoints using the permutation test available in the “exactRankTests” R package. The area under the curve (AUC) corresponds to the sum over time of all plasma soluble factor concentrations (cumulated concentration) or cell abundances (cumulated abundance) between H3 and D14. PP AUC and PB AUC were compared using the permutation test. Correlation analyses were performed using the Pearson coefficient. The density distributions of markers were compared using the Kolmogorov-Smirnov distance using CytoCompare^92^.

### Modeling

The linear regression model was constructed using SPADEVizR^20^. The abundance profiles of kinetic families were used as the entry parameter and IP-10 concentration as the biological value to predict. The validity of the model was assessed by excluding either one sample or one individual and by comparing predicted and observed values.

### Discrimination between post-prime and post-boost innate myeloid responses

The Least Absolute Shrinkage and Selection Operator (LASSO) approach was performed on R using the “lars” package. Centered and reduced abundance profiles of kinetic families were used as entry parameters. The validity of classification at each iteration was assessed by cross-validation. The best configuration was chosen as the lowest number of kinetic families used and the lowest error rate in cross-validation. Linear Discriminant Analysis (LDA) was performed on R with the “MASS” package based on the abundance profiles of kinetic families and classes (post-prime or post-boost) as entry parameters.

### Data availability and interactivity

Mass cytometry data were deposited publicly. FCS files are available on the FlowRepository database through ID FR-FCM-ZYBG, and the Cytobank platform under accession numbers 68443 and 68590. The http://data.idmitcenter.fr/MVA-innate-myeloid/ website provides interactive SPADE quality control data, interactive SPADE trees, interactive heatmaps, and interactive histograms. The interactive heatmaps avoid to juggle between Fig. 4 (heatmaps), Supplementary Figures S8–9 (phenotypic families abundance profiles), Fig. 5a (kinetic families), Table 1 (correspondance between phenotypic and kinetic families), and Fig. 7c (kinetic families LDA selection). They directly connect clusters and phenotypic families to their annotation, kinetic families, and their relevance as signature of the response to a first or a second immunization.

## Electronic supplementary material

Supplementary Information
