# Supplementary material for: Prime and Boost Vaccination Elicit a Distinct Innate Myeloid Cell Immune Response
Source: Sci Rep. 2018 Feb 15;8:3087. doi: 10.1038/s41598-018-21222-2 (PMC5814452; doi:10.1038/s41598-018-21222-2)
Supplement: Supplementary file 1 — Supplementary Information [file 41598_2018_21222_MOESM1_ESM.pdf]

Prime and Boost Vaccination Elicit a Distinct Innate Myeloid Cell Immune Response

Jean-Louis Palgen[1,2], Nicolas Tchitchek[1,2], Jamila Elhmouzi-Younes[1,2], Simon Delandre[1,2], Inana Namet[1,2], Pierre Rosenbaum[1,2], Nathalie Dereuddre-Bosquet[1,2], Frédéric Martinon[1,2], Antonio Cosma[1,2], Yves Levy[2,3], Roger Le Grand[1,2], Anne-Sophie Beignon[1,2,*]

[1] CEA – Université Paris Sud 11 – INSERM U1184, Immunology of Viral Infections and Autoimmune Diseases, IDMIT department, IBFJ, 92265 Fontenay-aux-Roses, France

[2] Vaccine Research Institute, Henri Mondor Hospital, 94010 Créteil, France

[3] Institut Mondor de Recherche Biomédicale – INSERM U955, équipe 16 physiopathologie et immunothérapies dans l'infection VIH, 94010, Créteil, France

Supplementary Information

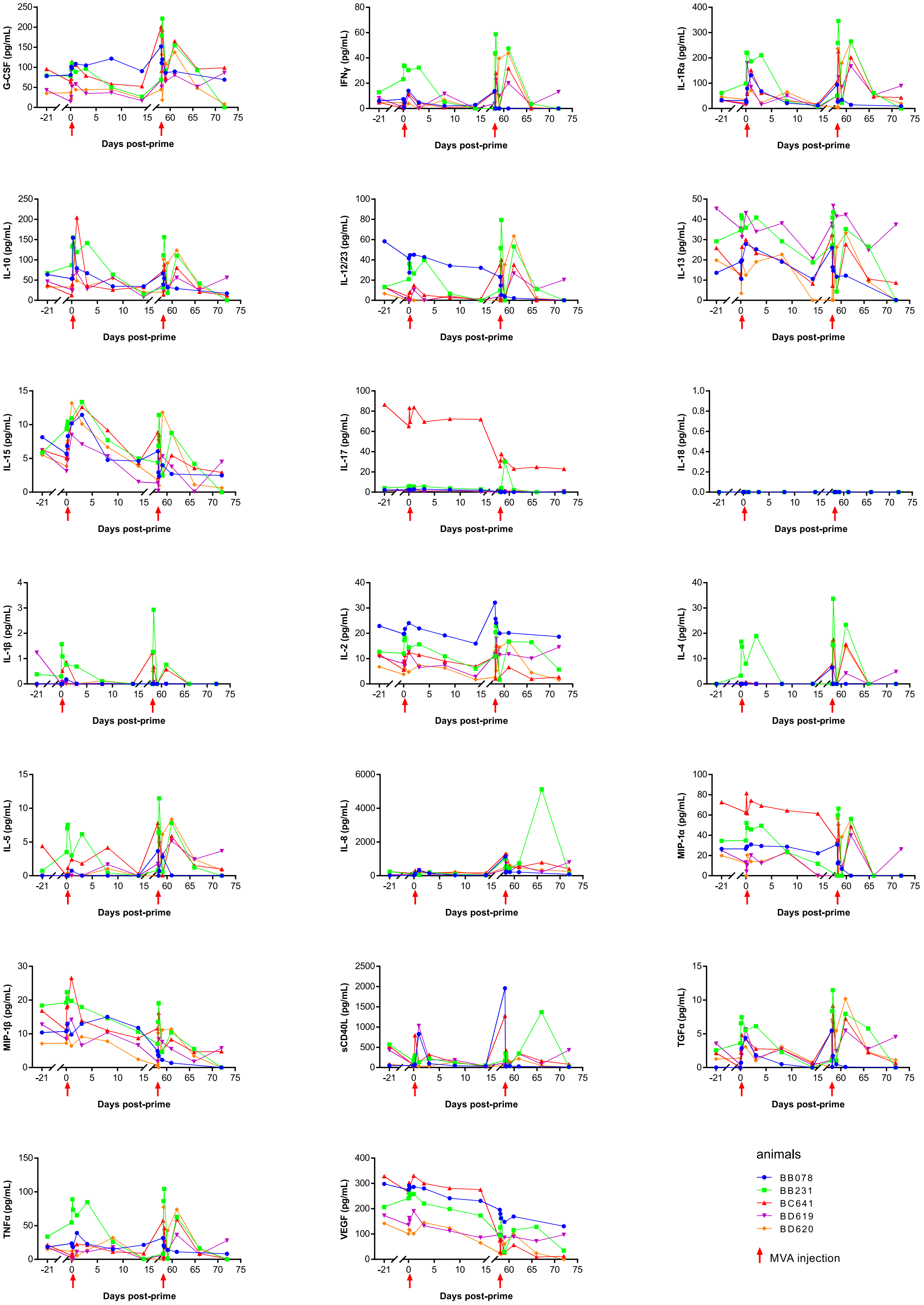
G-CSF (pg/mL)
IFNγ (pg/mL)
IL-1Ra (pg/mL)
IL-10 (pg/mL)
IL-12/23 (pg/mL)
IL-13 (pg/mL)
IL-15 (pg/mL)
IL-17 (pg/mL)
IL-18 (pg/mL)
IL-1β (pg/mL)
IL-2 (pg/mL)
IL-4 (pg/mL)
IL-5 (pg/mL)
IL-8 (pg/mL)
MIP-1α (pg/mL)
MIP-1β (pg/mL)
sCD40L (pg/mL)
TGFα (pg/mL)
TNFα (pg/mL)
VEGF (pg/mL)
Days post-prime
animals
BB078
BB231
BC641
BD619
BD620
MVA injection

Figure S1 – Kinetics of cytokine, chemokine, and growth factor concentrations not affected by vaccination. The blood levels of G-CSF, IFNγ, IL-1β, IL-1Rα, IL-10, IL-12_23, IL-13, IL-15, IL-17, IL-18, IL-2, IL-4, IL-5, IL-8, MIP-1α, MIP-1β, sCD40L, TGFα, TNFα, and VEGF are displayed. These cytokine levels were all assessed by Luminex. Cytokine concentrations below the limit of detection were set to 0. The concentrations had to be significantly different ($p \leq 0.01$) from the baseline level (H0PP) at two timepoints, at least, to be considered affected by vaccination However, IL-8 was not considered to be affected because of the significant difference in the concentrations between H0PP and H0PB, which was likely related to the Luminex assays performed independently for the PP and PB samples.

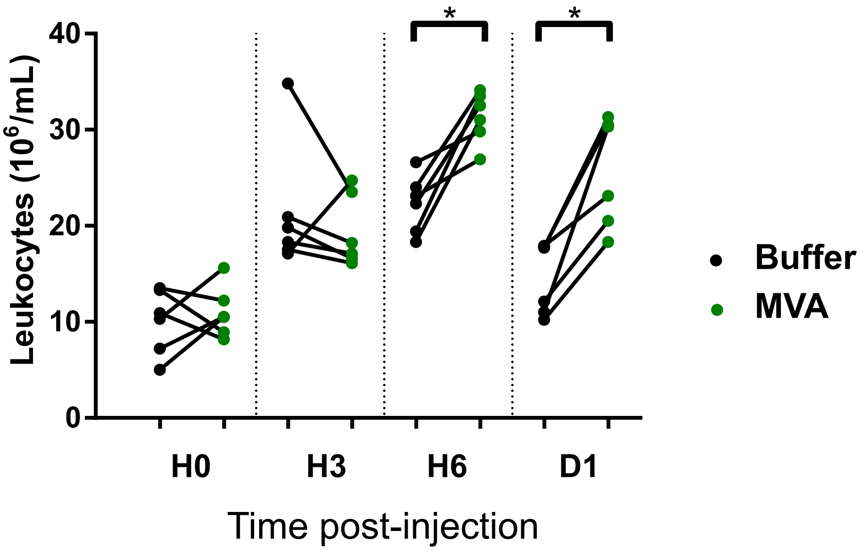

Figure S2. Impact of vaccine vs buffer subcutaneous injection. Six cynomolgus macaques were injected subcutaneously either with MVA HIV B or buffer only. Individual CBC are indicated. P-values were considered to be significant when $p<0.05$ using Wilcoxon test.

Figure available

-as pdf on FlowRepository database at:

https://flowrepository.org/id/RvFrm6T8FFIAXwRj85r3b8BMTHortPjRbJcbRyAzZgX63eQNcaDw2Tj8K8vM0694

-as interactive figure on IDMIT data dissemination platform at :

http://data.idmitcenter.fr/MVA-innate-myeloid/?page=qc

Figure S3 – Phenotype of each cell cluster. The distribution of all markers is displayed as histograms for each of the 600 clusters. The green color indicates that the distribution of the marker is unimodal and narrow (defined by a p-value below 0.05 for the unimodality test using Hartigan's dip test and an IQR below 2) and the red color indicates that at least one of the two conditions was not fulfilled. SPADE clustering markers are indicated in blue. The total number of cells per cluster, regardless of sample origin, is indicated between the parenthesis.

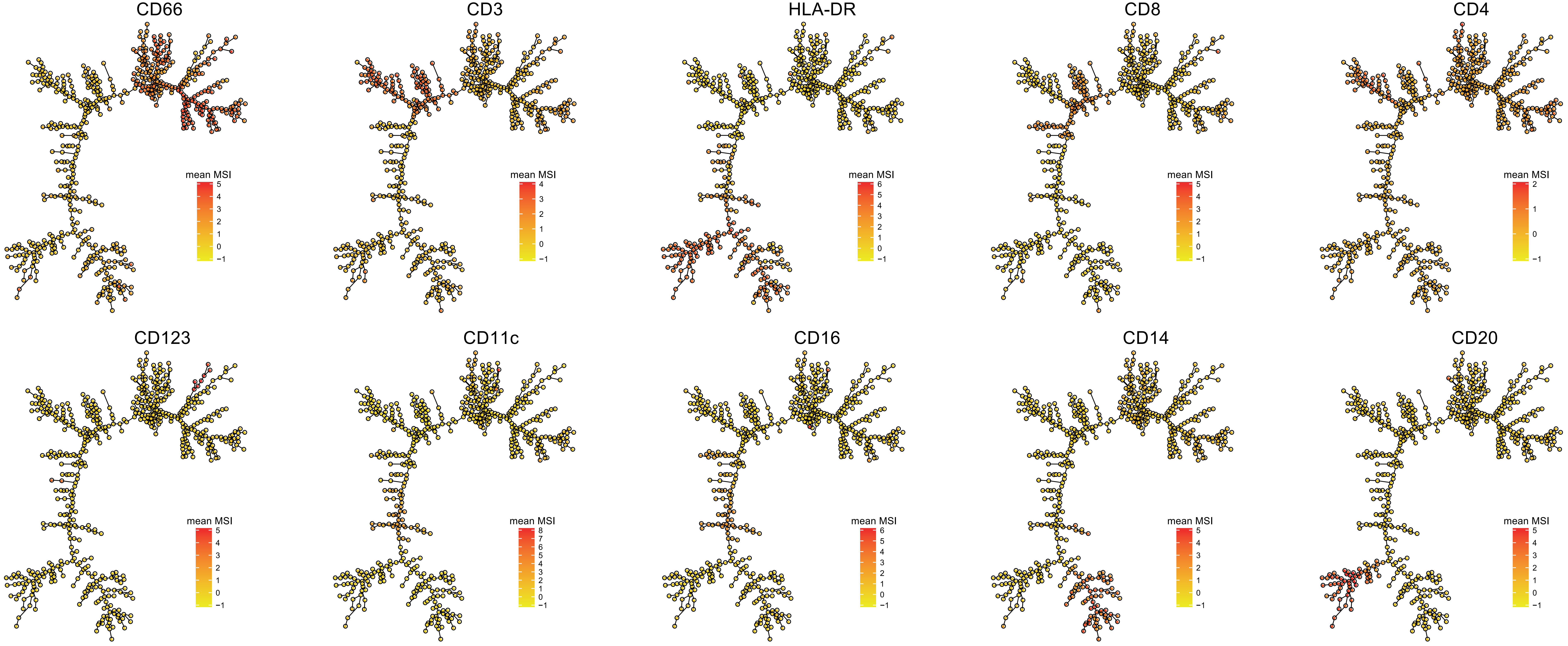
CD66
mean MSI
CD3
mean MSI
HLA-DR
mean MSI
CD8
mean MSI
CD4
mean MSI
CD123
mean MSI
CD11c
mean MSI
CD16
mean MSI
CD14
mean MSI
CD20
mean MSI

Figure S4 – Annotation of the SPADE tree. Each node of the SPADE tree is colored by the median expression of the indicated marker among all cells from the entire dataset. The size of the node is not proportional to the number of cells it contains. The scale is different for each marker.

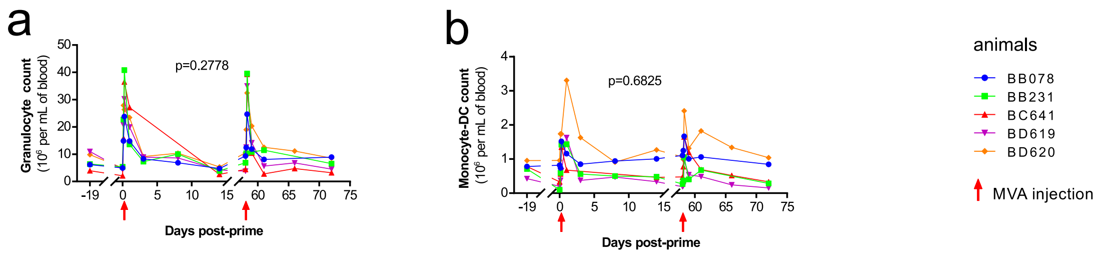

Figure S5 – Granulocytes and Monocytes-DC abundance profiles. The absolute blood counts of (**a**) granulocytes and (**b**) monocytes-DCs were quantified after MVA immunizations using CBC and mass cytometry data. Granulocytes and monocytes-DCs were defined as in **Figure 3c**. Granulocytes comprised neutrophils and basophils. Monocytes-DCs comprised monocytes, cDCs, pDCs, inflammatory cDCs/non-classical monocytes, and uncharacterized APCs. Individual abundance profiles are represented. Individual AUC after the prime (H3-D14PP) and boost (H3-D14PB) were calculated and compared using a permutation test. The mean PP and PB AUC, and p-values are indicated. P values were considered to be significant when $p \leq 0.01$. The red arrows indicate the timepoints of vaccination.

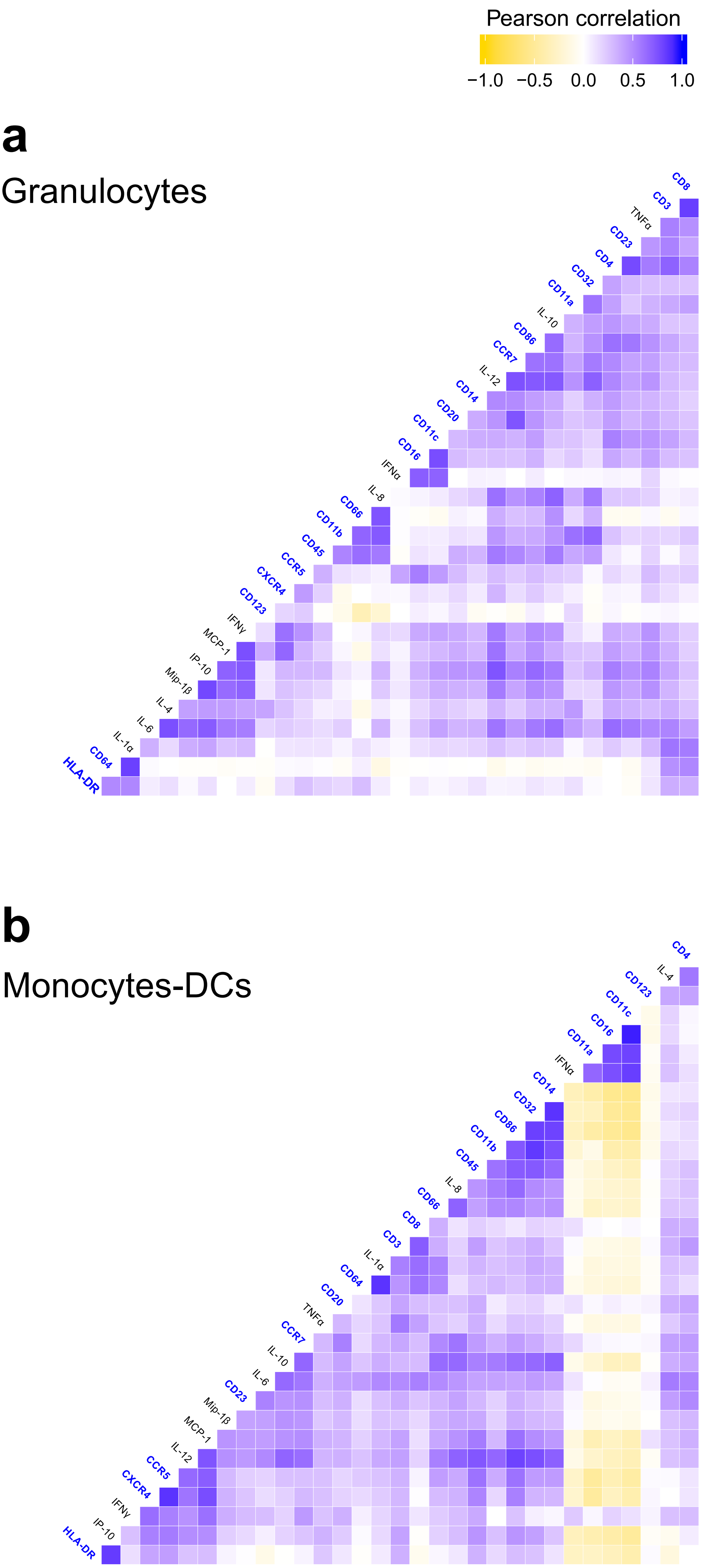
Pearson correlation
−1.0 −0.5 0.0 0.5 1.0
a
Granulocytes
HLA-DR
CD64
IL-1α
IL-6
IL-4
Mip-1β
IP-10
MCP-1
IFNγ
CD123
CXCR4
CCR5
CD45
CD11b
CD66
IL-8
IFNα
CD16
CD11c
CD20
CD14
IL-12
CCR7
CD86
IL-10
CD11a
CD32
CD4
CD23
TNFα
CD3
CD8
b
Monocytes-DCs
HLA-DR
IP-10
IFNγ
CXCR4
CCR5
IL-12
MCP-1
Mip-1β
CD23
IL-6
IL-10
CCR7
TNFα
CD20
CD64
IL-1α
CD3
CD8
CD66
IL-8
CD45
CD11b
CD86
CD32
CD14
IFNα
CD11a
CD16
CD11c
CD123
IL-4
CD4

Figure S6 – Correlations between marker expression. The Pearson coefficients of correlations between the expression of every marker are displayed for (**a**) granulocytes and (**b**) monocytes-DCs. Clustering markers are indicated in blue. Correlations were considered to be significant when $|R| \geq 0.75$.

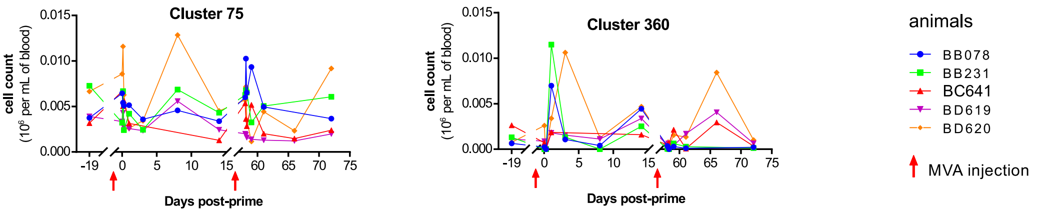

Figure S7 – Abundance profiles of the two pDC clusters. The individual number of cells/µL of blood for each pDC cluster is shown over time.

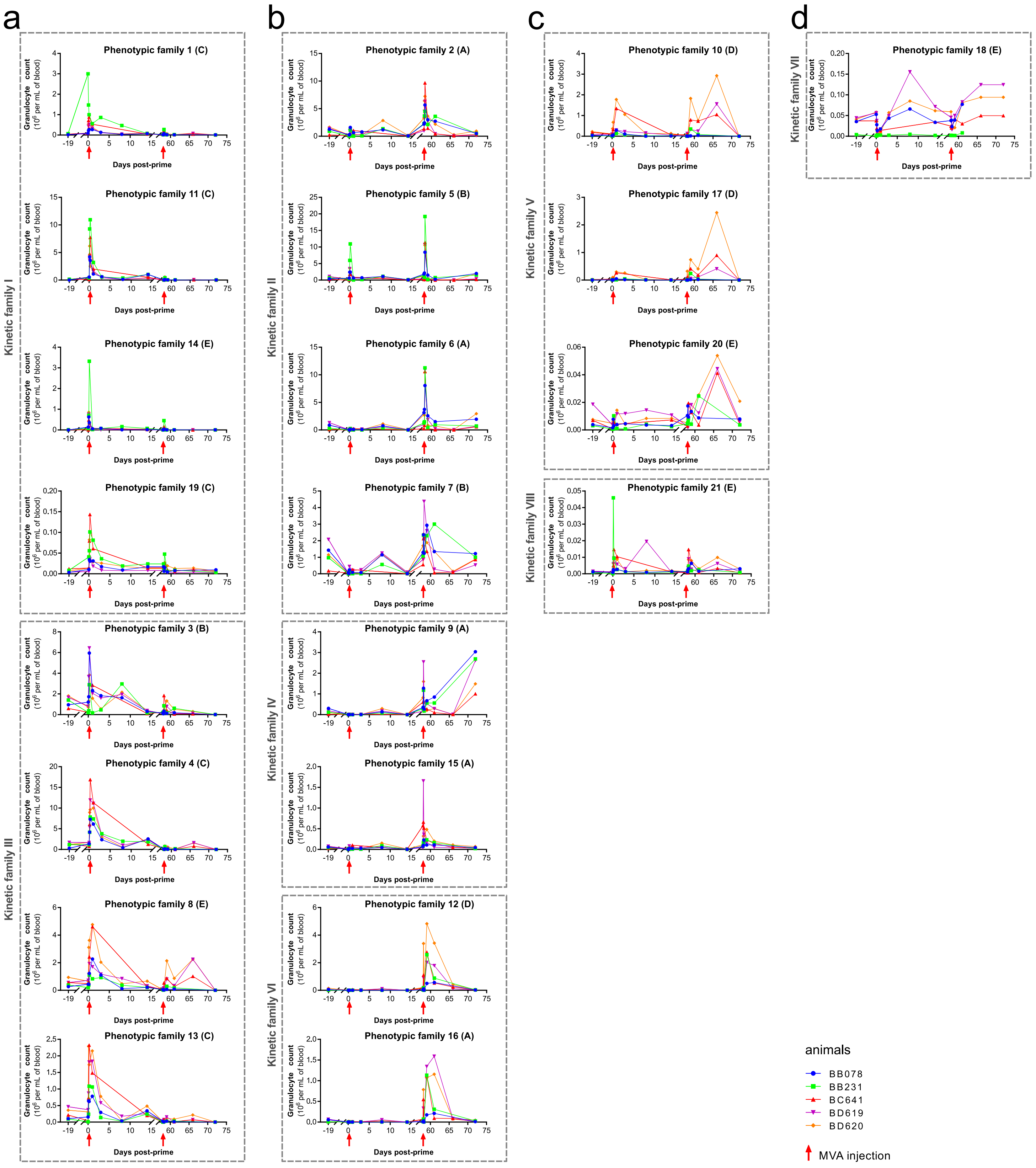

Figure S8 – Abundance profiles of the 21 granulocyte phenotypic families. The individual number of cells/µL of blood for each granulocyte phenotypic family is shown over time, the superfamily is indicated in parentheses. Phenotypic families were separated based on their abundance profiles: (**a**) post-prime enrichment, (**b**) post-boost enrichment, (**c**) both post-prime and post-boost enrichment, and (**d**) no or heterogeneous enrichment after each immunization. They were further grouped in dotted boxes depending on the kinetic family to which they belong, as defined in **Table 1**.

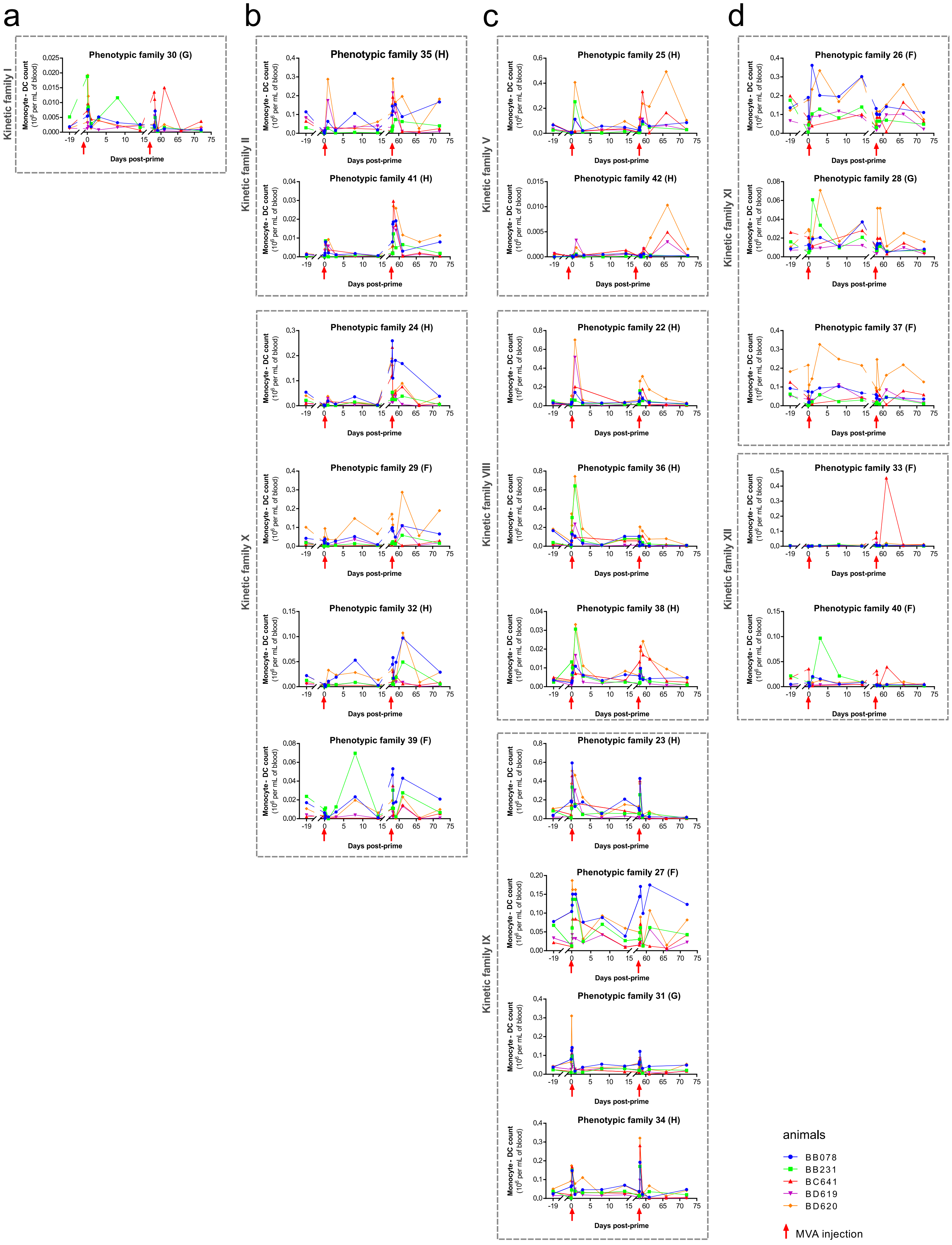
a
b
c
d
Kinetic family I
Phenotypic family 30 (G)
Kinetic family II
Phenotypic family 35 (H)
Phenotypic family 41 (H)
Kinetic family V
Phenotypic family 25 (H)
Phenotypic family 42 (H)
Kinetic family XI
Phenotypic family 26 (F)
Phenotypic family 28 (G)
Phenotypic family 37 (F)
Kinetic family X
Phenotypic family 24 (H)
Phenotypic family 29 (F)
Phenotypic family 32 (H)
Phenotypic family 39 (F)
Kinetic family VIII
Phenotypic family 22 (H)
Phenotypic family 36 (H)
Phenotypic family 38 (H)
Kinetic family XII
Phenotypic family 33 (F)
Phenotypic family 40 (F)
Kinetic family IX
Phenotypic family 23 (H)
Phenotypic family 27 (F)
Phenotypic family 31 (G)
Phenotypic family 34 (H)
Monocyte - DC count
(10^6 per mL of blood)
Days post-prime
animals
BB078
BB231
BC641
BD619
BD620
MVA injection

Figure S9 – Abundance profiles of the 21 monocyte-DC phenotypic families. The individual number of cells/µL of blood for each monocyte-DC phenotypic family is shown over time, the superfamily is indicated in parentheses. Phenotypic families were separated based on their abundance profiles: (**a**) post-prime enrichment, (**b**) post-boost enrichment, (**c**) both post-prime and post-boost enrichment, and (**d**) no or heterogeneous enrichment after each immunization. They were further grouped in dotted boxes depending on the kinetic family to which they belong, as defined in **Table 1**.

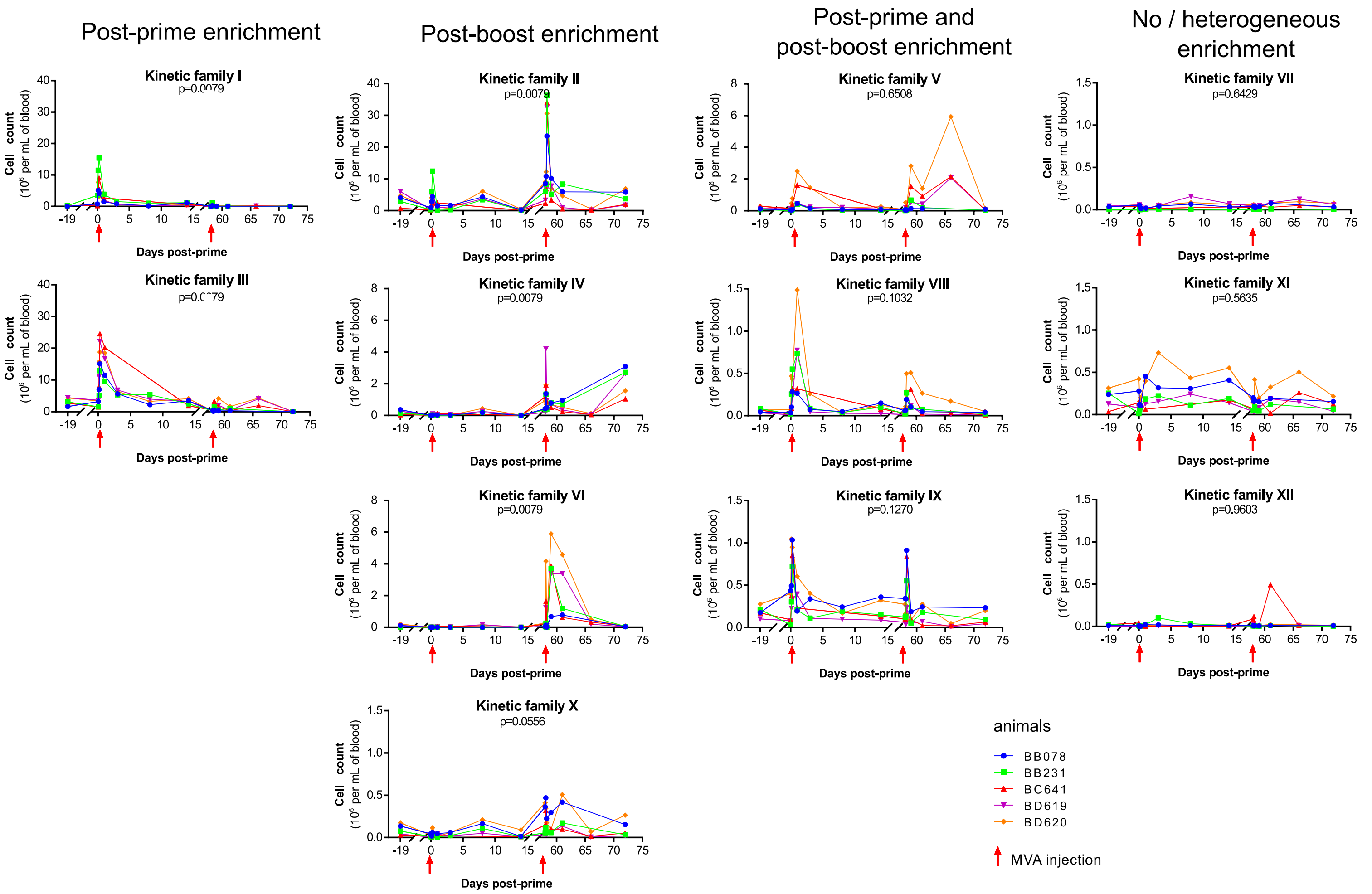

Figure S10 – Abundance profiles of kinetic families. The kinetics of the twelve kinetic families defined on **Figure 5a**, are given at the individual level. They are regrouped into kinetic patterns displaying enrichment essentially post-prime, essentially post-boost, both after the prime and boost, or no or heterogeneous enrichment after each immunization. As for **Figure 5a**, the individual AUC after the prime (H3-D14PP) and boost (H3-D14PB) were calculated for each kinetic family and compared using a permutation test. The p-values are indicated and considered to be significant when p≤0.01. Note that the scale of the Y-axis is specific to each kinetic family. The red arrows indicate the prime and boost injections.

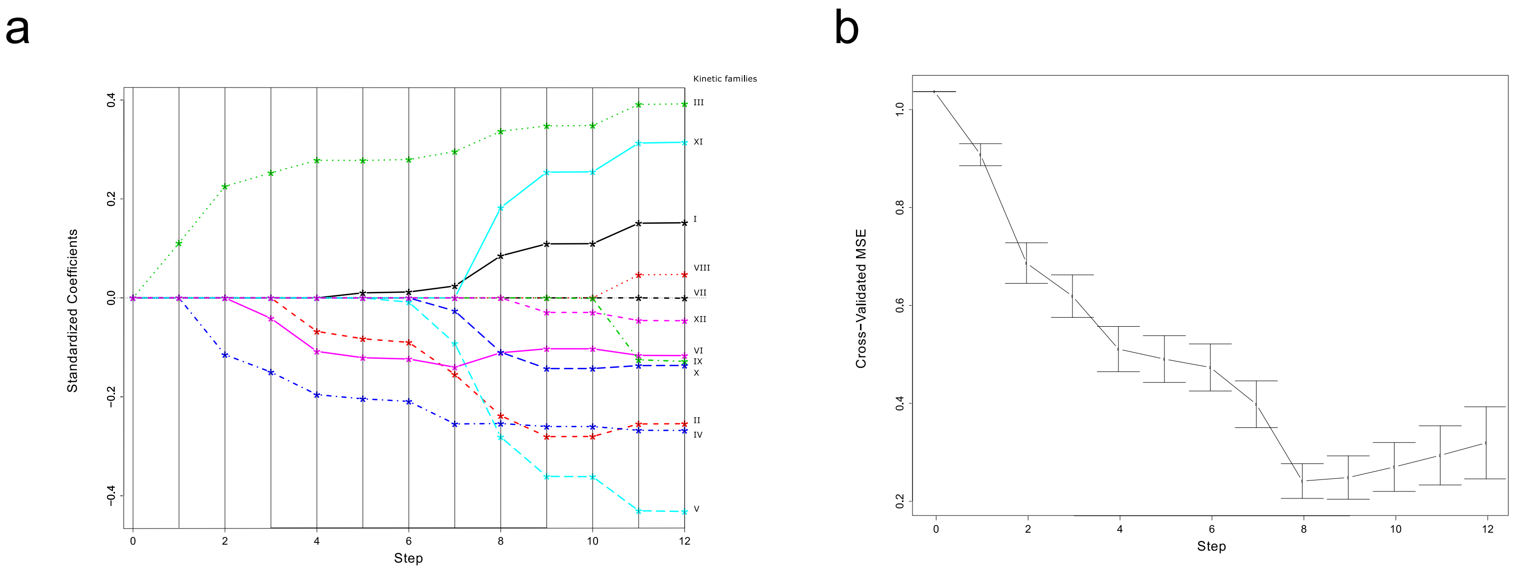

Figure S11 – Selection of kinetic families for LDA classification using LASSO. (**a**) The evolution of LDA-LASSO coefficients at each step for each kinetic family is represented. (**b**) The evolution of the mean standard error (MSE) at each step assessed by leave-one-out cross-validation is shown.

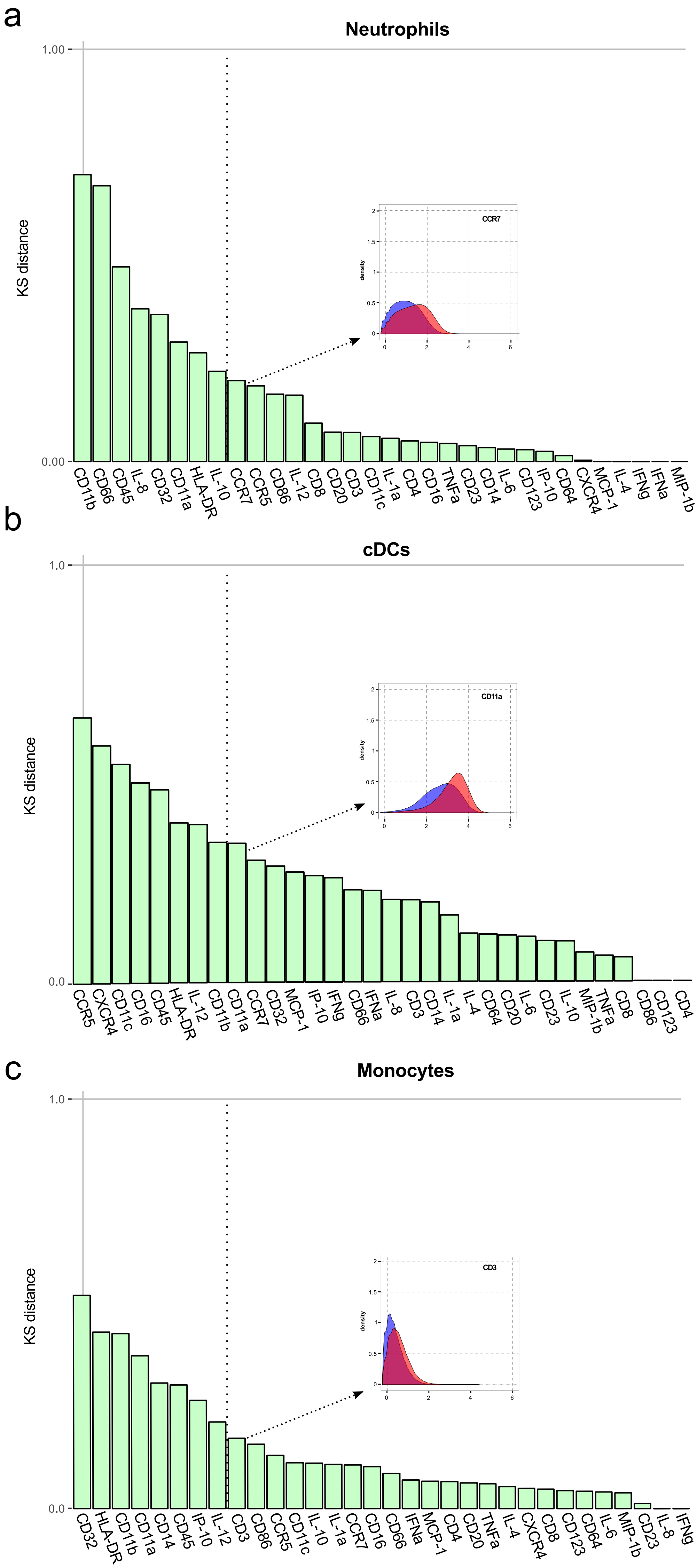
a
Neutrophils
KS distance
1.00
0.00
CD11b CD66 CD45 IL-8 CD32 CD11a HLA-DR IL-10 CCR7 CCR5 CD86 IL-12 CD8 CD20 CD3 CD11c IL-1a CD4 CD16 TNFa CD23 CD14 IL-6 CD123 IP-10 CD64 CXCR4 MCP-1 IL-4 IFNg IFNa MIP-1b
CCR7
density
b
cDCs
KS distance
1.0
0.0
CCR5 CXCR4 CD11c CD16 CD45 HLA-DR IL-12 CD11b CD11a CCR7 CD32 MCP-1 IP-10 IFNg CD66 IFNa IL-8 CD3 CD14 IL-1a IL-4 CD64 CD20 IL-6 CD23 IL-10 MIP-1b TNFa CD8 CD86 CD123 CD4
CD11a
density
c
Monocytes
KS distance
1.0
0.0
CD32 HLA-DR CD11b CD11a CD14 CD45 IP-10 IL-12 CD3 CD86 CCR5 CD11c IL-10 IL-1a CCR7 CD16 CD66 IFNa MCP-1 CD4 CD20 TNFa IL-4 CXCR4 CD8 CD123 CD64 IL-6 MIP-1b CD23 IL-8 IFNg
CD3
density

Figure S12 – Comparison of marker distribution between cells that best discriminate between the post-prime and post-boost innate myeloid response. The Kolmogorov-Smirnov distance was computed to compare each marker distribution between prime- and boost-enriched neutrophils (**a**), cDCs (**b**), and monocytes (**c**). The prime cell signature consisted of cells from kinetic families III, XI, and I, whereas the boost cell signature consisted of cells from kinetic families V, II, IV, X, VI, and I as defined in **Figure 7c** after LASSO/LDA. For each category (prime-enriched or boost-enriched), the histogram was plotted using all cells (all animals and all timepoints) belonging to the indicated kinetic families and the indicated cellular compartment. However, there were a few exceptions. Since only CD14^{low} monocytes were found associated with the prime (**Table 1**), all monocytes that were not exclusively associated with the boost were used for the comparison of marker expression (*i.e.* monocytes from kinetic families V, VIII, IX, and not from kinetic families II, IV, VI and X). In addition, neither inflammatory cDCs/non-classical monocytes (monocyte-DC phenotypic family 11) nor uncharacterized APCs (monocyte phenotypic families 9 and 10) were included with the cDCs or monocytes. The dotted line indicates the top eight markers, with the highest KS distance, shown in **Figure 7**. The histogram for the ninth highest KS distance is indicated for each population.

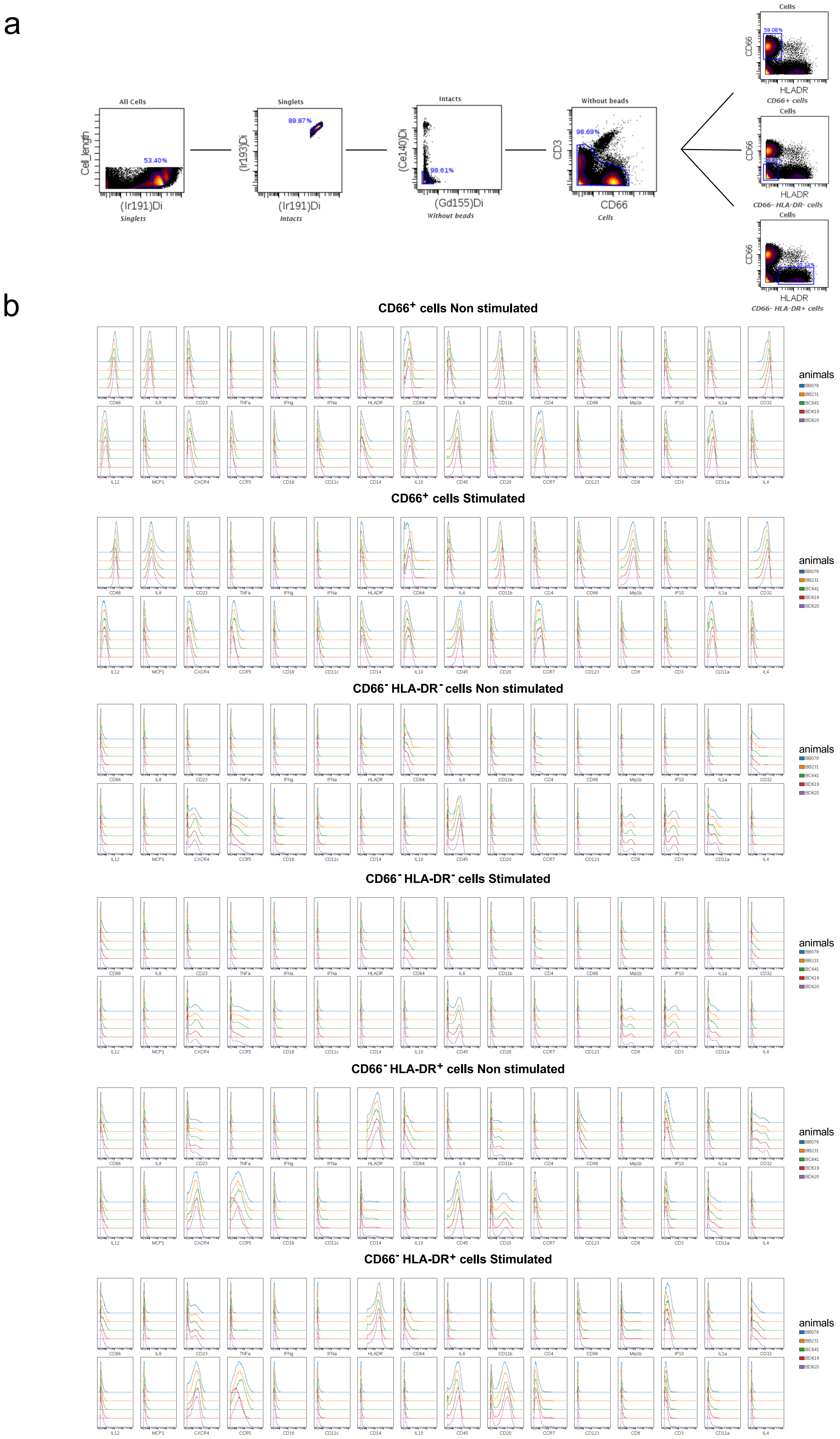
a
All Cells
Cell_length
53.40%
(Ir191)Di
Singlets
Singlets
(Ir193)Di
89.87%
(Ir191)Di
Intacts
Intacts
(Ce140)Di
98.61%
(Gd155)Di
Without beads
Without beads
CD3
98.69%
CD66
Cells
Cells
CD66
59.08%
HLADR
CD66+ cells
Cells
CD66
HLADR
CD66- HLA-DR- cells
Cells
CD66
HLADR
CD66- HLA-DR+ cells
b
CD66+ cells Non stimulated
CD66+ cells Stimulated
CD66- HLA-DR- cells Non stimulated
CD66- HLA-DR- cells Stimulated
CD66- HLA-DR+ cells Non stimulated
CD66- HLA-DR+ cells Stimulated
CD66 IL8 CD23 TNFa IFNg IFNa HLADR CD64 IL6 CD11b CD4 CD86 Mip1b IP10 IL1a CD32
IL12 MCP1 CXCR4 CCR5 CD16 CD11c CD14 IL10 CD45 CD20 CCR7 CD123 CD8 CD3 CD11a IL4
animals
BB078
BB231
BC641
BD619
BD620

Figure S13 – Staining and acquisition controls. The same two control samples were stained and acquired simultaneously for each of the five stainings and acquisitions of samples from the five vaccinated animals. These control samples were obtained after *ex vivo* restimulation of whole blood from one unrelated macaque with or without a TLR ligand cocktail (TLR 3, 4, 7, and 8) composed of 1 µg/mL LPS (from *E. coli* 0111:B4, Invivogen), 100 µg/mL Poly I-C (Invivogen), and 10 µg/mL R848 (Mabtech). Whole blood was incubated with or without stimulation for 6 h, with the addition of 10 µg/mL BFA (Sigma) during the last 4 h. The cells were then fixed, red-blood cells lysed, and frozen as vaccinated animal samples. (**a**) Gating strategy to define the cell populations to compare staining profiles between control samples. The non stimulated control sample for the acquisition of macaque BB078 samples is shown. (**b**) Distribution of all markers across $CD66^{+}$, $CD66^{-}$ $HLA\text{-}DR^{-}$, and $CD66^{-}$ $HLA\text{-}DR^{+}$ cells. Each control samples acquisition is labelled with the ID of the macaque samples that were stained and acquired simultaneously.

	Baseline vs H3PP	Baseline vs H6PP	Baseline vs D1PP	Baseline vs D3PP	Baseline vs D8PP	Baseline vs D14PP
CRP	nd	nd	0.0079	0.0159	nd	nd
G-CSF	0.7698	0.1508	0.1746	0.4841	0.8333	0.5238
IFNg	1.0000	0.8254	0.3413	0.8254	0.8492	0.1270
IL-10	0.7222	0.0635	0.0952	0.6032	0.7698	0.2460
IL-12_23 (p40)	0.9206	0.7937	0.6746	0.9365	0.8651	0.5635
IL-13	1.0000	0.5159	0.3889	0.3889	0.6190	0.1667
IL-15	0.4048	0.2143	0.0159	0.0159	0.3651	0.3413
IL-17	0.8810	1.0000	0.8810	1.0000	1.0000	1.0000
IL-18	1.0000	1.0000	1.0000	1.0000	1.0000	1.0000
IL-1b	1.0000	0.2857	0.2857	1.0000	1.0000	1.0000
IL-1Ra	0.9603	0.0952	0.0238	0.5635	1.0000	0.0714
IL-2	0.6587	0.5794	0.5397	0.5397	0.9048	0.4683
IL-4	1.0000	1.0000	0.7222	1.0000	1.0000	1.0000
IL-5	1.0000	0.5635	0.6825	0.7222	0.4841	1.0000
IL-6	0.0476	0.0079	0.0079	0.1667	1.0000	0.1667
IL-8 (CXCL8)	0.1270	0.0556	0.0397	0.0159	0.3175	0.9127
IP-10† (CXCL10)	0.0079	0.0159	0.0079	0.0238	0.0952	0.3968
MCP-1 (CCL2)	0.0079	0.0079	0.0079	0.0079	0.0952	1.0000
MIP-1a (CCL3)	1.0000	0.8095	0.7222	0.8413	0.9048	0.5952
MIP-1b (CCL4)	0.4841	0.3016	0.3175	0.9286	1.0000	0.4127
sCD40L	1.0000	0.2698	0.1270	0.1746	0.2143	0.0714
TGFa	0.4444	0.0714	0.0159	0.2381	0.2698	0.4841
TNFa	0.8571	0.7460	0.6032	0.6032	1.0000	0.3730
VEGF	0.6587	0.7143	0.6667	0.8333	0.8254	0.5556

	Baseline vs H0PB	Baseline vs H3PB	Baseline vs H6PB	Baseline vs D1PB	Baseline vs D3PB	Baseline vs D8PB	Baseline vs D14PB
CRP	nd	nd	nd	0.0079	nd	nd	nd
G-CSF	0.1905	0.0635	0.1111	0.1190	0.0238	0.2778	1.0000
IFNg	0.9286	0.4444	0.3571	0.8810	0.0952	0.2778	0.4841
IL-10	0.9127	0.6111	0.2302	0.9127	0.1905	0.6349	0.2063
IL-12_23 (p40)	1.0000	0.5079	0.3730	0.6984	0.1905	0.5476	0.4048
IL-13	0.8095	0.6984	0.8571	0.7222	0.3889	0.4762	0.1984
IL-15	0.7063	0.4921	1.0000	1.0000	0.9603	0.0556	0.0794
IL-17	0.7222	0.7222	0.8810	1.0000	0.7222	0.8810	0.7222
IL-18	1.0000	1.0000	1.0000	1.0000	1.0000	1.0000	1.0000
IL-1b	1.0000	0.4444	0.4444	1.0000	0.1667	1.0000	1.0000
IL-1Ra	0.5000	0.3413	0.2381	0.6111	0.0476	0.6032	0.6349
IL-2	0.6111	0.3492	0.1508	1.0000	0.2937	0.6746	0.7937
IL-4	0.4444	0.4444	0.1667	1.0000	0.0476	1.0000	1.0000
IL-5	0.2063	0.2063	0.0873	0.3254	0.0476	0.2063	0.6825
IL-6	0.4444	0.2857	0.0079	0.4444	0.6111	0.2857	0.2857
IL-8 (CXCL8)	0.0079	0.0079	0.0079	0.0079	0.0079	0.0476	0.0794
IP-10† (CXCL10)	0.0238	0.0079	0.0397	0.0079	0.6032	0.7460	0.1905
MCP-1 (CCL2)	0.4127	0.0317	0.0079	0.2778	0.1190	0.4127	0.6190
MIP-1a (CCL3)	0.2540	0.8651	0.8651	0.1429	0.5238	0.0238	0.0635
MIP-1b (CCL4)	0.0714	0.2698	0.7143	0.0794	0.3333	0.0238	0.0079
sCD40L	0.2857	0.7063	0.2937	0.8651	0.0476	0.2857	0.9286
TGFa	0.3095	0.2698	0.0635	0.5238	0.0556	0.1429	1.0000
TNFa	1.0000	0.4524	0.5159	0.6587	0.0873	0.5238	0.3889
VEGF	0.0714	0.0794	0.0952	0.0317	0.0635	0.0476	0.0397

	H3PP vs H3PB	H6PP vs H6PB	D1PP vs D1PB	D3PP vs D3PB	D8PP vs D8PB	D14PP vs D14PB
CRP	nd	nd	0.0476	0.2778	nd	nd
G-CSF	0.1270	0.3889	0.9048	0.0635	0.6190	0.6746
IFNg	0.4444	0.4048	0.5635	0.1270	0.1905	1.0000
IL-10	1.0000	0.3730	0.0635	0.6032	0.1429	0.8571
IL-12_23 (p40)	0.5079	0.5635	0.3889	0.2937	0.8651	1.0000
IL-13	0.7778	0.8968	0.2857	0.8492	0.2143	0.9206
IL-15	0.1905	0.4603	0.0397	0.0397	0.0079	0.1429
IL-17	0.7222	0.8810	0.8810	0.7222	1.0000	1.0000
IL-18	1.0000	1.0000	1.0000	1.0000	1.0000	1.0000
IL-1b	1.0000	0.8810	0.1667	0.2857	0.2857	1.0000
IL-1Ra	0.4048	0.8651	0.2143	0.1587	0.2063	0.3016
IL-2	0.6984	0.4444	0.4683	0.7222	0.5794	0.7619
IL-4	1.0000	0.2857	0.7222	0.2460	1.0000	1.0000
IL-5	0.3651	0.3571	0.3571	0.1349	0.7460	0.1667
IL-6	0.9286	0.9365	0.0238	0.2460	0.4444	1.0000
IL-8 (CXCL8)	0.0079	0.0556	0.2698	0.0238	0.0476	0.0714
IP-10† (CXCL10)	0.0079	0.0079	0.8571	0.0952	0.1587	0.0794
MCP-1 (CCL2)	0.1905	0.7063	0.0556	0.0873	0.3016	0.7143
MIP-1a (CCL3)	0.7381	1.0000	0.0794	0.7778	0.0079	0.4048
MIP-1b (CCL4)	0.0794	0.2619	0.0556	0.1111	0.0159	0.0397
sCD40L	0.6746	0.7778	0.1270	0.3175	0.5476	0.8651
TGFa	0.6667	0.3571	0.0635	0.1429	0.5159	0.6905
TNFa	0.8810	0.7222	0.2778	0.4048	0.1111	1.0000
VEGF	0.0556	0.0476	0.0238	0.0397	0.0476	0.0952

Table S1 – Statistics for plasma soluble factor concentrations. P-values after the permutation test for the indicated comparisons (all timepoints against baseline and homologous PP and PB timepoints against each other) are shown and colored in red when ≤0.01. H0PP was chosen as the baseline for all soluble factors, except IP-10, as indicated with †, for which D-21PP was used as the baseline because macaque BD619 showed an aberrantly high level of IP-10 concentration at H0PP. nd, not determined. The areas under the curve (H3-D14 PP *vs.* H3-D14 PB) were computed giving the same weight to each timepoint, irrespective of the actual linear time scale (*e.g.* abundance at H3 has the same weight as abundance at D8) and compared using the permutation test. A permutation test, previously used to compare cytometry data[22], was adapted to compare soluble factor levels, cell abundances, and the corresponding areas under the curve, because our dataset contained paired samples with n=5 and missing datapoints.

	H0PP vs H3PP	H0PP vs H6PP	H0PP vs D1PP	H0PP vs D3PP	H0PP vs D8PP	H0PP vs D14PP	H0PP vs H0PB	H0PP vs H3PB	H0PP vs H6PB	H0PP vs D1PB	H0PP vs D3PB	H0PP vs D8PB	H0PP vs D14PB	H3PP vs H3PB	H6PP vs H6PB	D1PP vs D1PB	D3PP vs D3PB	D8PP vs D8PB	D14PP vs D14PB
Leukocytes	0.0079	0.0079	0.0079	0.0238	0.0159	0.5317	0.2937	0.0079	0.0079	0.0079	0.0556	0.0893	0.3254	0.0794	0.4365	0.0556	0.8571	0.5143	0.6746
Granulocytes	0.0079	0.0079	0.0079	0.0079	0.0079	0.8413	0.1190	0.0079	0.0079	0.0079	0.1111	0.1607	0.1349	0.0397	0.6508	0.0794	0.9048	0.5143	0.1429
Monocytes-DCs	0.3175	0.0238	0.0397	0.2540	0.4365	0.4286	0.5794	0.4444	0.0238	0.1508	0.2222	0.5536	0.7540	1.0000	0.5476	0.1349	0.7937	1.0000	0.4841

Table S2 – Statistics for absolute blood leukocyte, granulocyte, and monocyte-DC counts. P-values after the permutation test for the indicated comparisons are shown and colored in red when ≤0.01.

Metal	Marker	Clone	Surface	Intra-cellular
141Pr	CD66	TET2	●	
142Nd	HLA-DR	L243	●	
143Nd	CD3	SP34.2	●	
144Nd	CD64	10.1	●	
145Nd	CD8	RPAT8	●	
146Nd	IL-6	MQ2.13A5		●
147Sm	CD123	7G3	●	
148Nd	IL-4	8D48		●
149Sm	CD11a	HI111	●	
150Nd	CD11b	ICRF144	●	
151Eu	IL-8	G265.8		●
152Sm	CD4	L200	●	
153Eu	CD23	9P25	●	
154Sm	CD86	IT2.2	●	
156Gd	MIP-1b	D21-1351		●
158Gd	IP-10	6D4		●
159Tb	TNFa	MAb11		●
160Gd	IL-1a	364/3B3		●
161Dy	CD32	FLI8.26	●	
162Dy	IL-12	C8.6		●
163Dy	MCP-1	2H5		●
164Dy	CXCR4	12G5	●	
165Ho	IFNg	B27		●
166Er	CCR5	3A9	●	
167Er	CD16	3G8	●	
168Er	CD11c	3.9	●	
169Tm	IFNa	LT27/295		●
170Er	CD14	M5E2	●	
171Yb	IL-10	127107		●
172Yb	CD45	D058-1283	●	
174Yb	CD20	2H7	●	
175Lu	CCR7	G043H7	●	

Table S3 – Mass cytometry antibody panel. The associated clone and metal are indicated for each marker. The right columns indicate whether the markers were extra- or intra-cellularly stained. [∞]The TET2 antibody clone recognizes four members of the CD66 family: CD66a (CEACAM1), CD66b (CEACAM8), CD66c (CEACAM6), and CD66e (CEACAM5). [§]The FLI8.26 antibody clone reacts with CD32a and CD32b. [‡]The LT27/295 antibody clone stains most IFNa subtypes, but not IFNa 2b.

	H0PP vs H3PP	H0PP vs H6PP	H0PP vs D1PP	H0PP vs D3PP	H0PP vs D8PP	H0PP vs D14PP	H0PP vs H0PB	H0PP vs H3PB	H0PP vs H6PB	H0PP vs D1PB	H0PP vs D3PB	H0PP vs D8PB	H0PP vs D14PB	H3PP vs H3PB	H6PP vs H6PB	D1PP vs D1PB	D3PP vs D3PB	D8PP vs D8PB	D14PP vs D14PB
Kinetic Family I	0.0079	0.0238	0.1667	1.0000	0.5635	0.9524	0.0079	0.0476	0.7222	0.0079	0.0079	0.3750	0.0079	0.0079	0.0079	0.0079	0.0079	0.6286	0.0079
Kinetic Family II	0.0079	0.0079	0.0794	0.1825	0.0079	0.2302	0.0079	0.0079	0.0079	0.0079	0.1190	0.5357	0.0079	0.0159	0.0079	0.0079	0.1429	0.0286	0.0079
Kinetic Family III	0.0079	0.0079	0.0079	0.0079	0.1984	0.5556	0.0079	0.0079	0.1905	0.3492	0.0238	0.4464	0.0079	0.0079	0.0079	0.0079	0.0079	0.8000	0.0079
Kinetic Family IV	0.5635	0.0238	0.0873	0.1825	0.0079	0.6825	0.0079	0.0079	0.0079	0.0079	0.0079	0.0179	0.0079	0.0079	0.0079	0.0079	0.0159	0.0857	0.0079
Kinetic Family V	0.9524	0.3016	0.0079	0.0794	0.4921	0.5556	0.2619	0.4127	0.5397	0.0476	0.0794	0.0179	0.5714	0.6032	0.4206	1.0000	0.8413	0.0286	0.3730
Kinetic Family VI	0.8810	0.3333	0.1190	0.3571	0.1667	0.8968	0.0079	0.0476	0.0079	0.0079	0.0079	0.0179	0.0079	0.0476	0.1190	0.0079	0.0317	0.0286	0.0079
Kinetic Family VII	0.2460	0.0476	0.0556	0.9048	0.2381	1.0000	0.6190	0.3889	0.0952	0.5873	0.4444	0.1071	1.0000	0.9048	0.1984	0.0952	0.4286	0.8857	1.0000
Kinetic Family VIII	0.0079	0.0079	0.0079	0.0556	0.6190	0.0476	0.6508	0.1667	0.0079	0.0079	0.2778	0.3393	0.7937	0.1429	0.4921	0.0714	0.5556	0.8286	0.0317
Kinetic Family IX	0.1587	0.0238	0.3968	0.7460	0.8254	1.0000	0.9206	0.8571	0.0317	0.4127	0.6667	0.2321	0.4603	0.0317	0.6667	0.0317	0.4603	0.0571	0.3254
Kinetic Family X	1.0000	0.1270	0.3016	0.2063	0.0159	0.8254	0.0476	0.0159	0.0317	0.0079	0.0079	0.7857	0.0476	0.0159	0.1984	0.1667	0.0556	0.0857	0.2063
Kinetic Family XI	0.3651	0.4603	0.6111	0.2302	0.5397	0.4683	0.3492	0.2857	1.0000	0.5000	0.8651	0.4643	0.4444	0.9206	0.4048	0.3333	0.1429	0.7714	0.0794
Kinetic Family XII	0.0159	0.0317	0.8413	0.7222	0.6905	0.8175	0.9603	0.6429	0.3571	0.2143	1.0000	0.8929	0.6190	0.0079	0.0952	0.2222	1.0000	0.5143	0.7540

Table S4 – Statistics for kinetic family abundance. P-values after the permutation test for the indicated comparisons are shown and colored in red when ≤0.01.

	BB078	BB231	BC641	BD619	BD620	ALL
**D-19PP**	114 244	117 463	147 515	160 415	137 862	677 499
**D0PP**	105 318	62 451	71 336	131 117	161 546	531 768
**H3PP**	158 371	159 250	190 509	133 164	194 991	836 285
**H6PP**	154 642	197 605	192 414	186 113	150 724	881 498
**D1PP**	146 185	87 666	153 658	151 935	185 683	725 127
**D3PP**	115 397	93 663		134 085	132 034	475 179
**D8PP**	133 512	124 869		121 685	97 003	477 069
**D14PP**	157 067	146 843	144 849	116 936	144 753	710 448
**D0PB**	173 286	200 907	68 326	147 323	139 075	728 917
**H3PB**	214 595	164 924	68 369	64 272	113 498	625 658
**H6PB**	225 773	244 110	270 132	233 035	233 264	1 206 314
**D1PB**	193 136	128 369	234 244	159 883	175 616	891 248
**D3PB**	108 404	189 688	95 487	103 584	164 337	661 500
**D8PB**	226 125		239 341	233 747	318 350	1 017 563
**D14PB**	225 733	230 585	140 196	150 390	295 739	1 042 643
**TOTAL**	2 451 788	2 148 393	2 016 376	2 227 684	2 644 475	11 488 716

Table S5 – Numbers of cells acquired for each sample. The number of cells of interest acquired with the CyTOF device (live non $CD3^+CD66^+$ singlets) is displayed for each animal and each timepoint. Baseline samples were collected 19 days before the prime (D-19) or just before the prime (H0PP). Samples were also collected just before the boost, 58 days after the prime (H0PB). The sums of all cells acquired for each animal and each timepoint are indicated at the bottom and on the right, respectively. Missing samples are indicated in gray. The smallest sample (macaque BB231 at D0PP) contained 62,451 cells, whereas the largest sample (macaque BD620 at D8PB) contained 318,350 cells. Samples from macaque BC641 at D3PP and D8PP and macaque BB231 at D8PB were excluded from the SPADE analysis due to the low number of acquired cells of interest (<50,000 live non $CD3^+$ $CD66^+$ singlets). The SPADE analysis was performed on 72 samples for a total of 11,488,716 cells (**Supplementary Table S5**). We avoided a bias in the analysis toward samples with significantly more cells than others by performing random pre-downsampling by selecting the same number of cells (60,000) in each sample. All cells were finally up-sampled in the last SPADE step.

Markers	Number of non-uniform clusters	Percentage of non-uniform clusters
CD64	150	25
CD45	36	6
CD32	30	5
CD11a	13	2.17
CD4	8	1.33
CD8	8	1.33
CD11b	7	1.17
CD11c	4	0.67
CD123	4	0.67
CD20	4	0.67
CD66	4	0.67
CD3	3	0.50
CXCR4	3	0.50
CD16	2	0.33
HLA-DR	2	0.33
CCR5	1	0.17
CD14	1	0.17
CCR7	0	0
CD23	0	0
CD86	0	0

Table S6 – Cluster uniformity per marker. The number and corresponding percentage of 'non-uniform' clusters are shown for each marker. Non-uniform clusters were defined as clusters with non-unimodal or non-narrow expression for the indicated marker (non-unimodality being defined with the Hartigan's dip test using a p-value threshold of 0.05 and non-narrow expression by an IQR ≥ 2). 62.5% of the clusters (375 of 600) showed a good SPADE clustering. Imperfect clustering quality was mainly related to the bimodal distribution of CD64 in 25% of the clusters (**Supplementary Figure S2** and **Table S6**). Except for CD64, 97% of the clusters were uniform for the expression of at least 18 of the 19 remaining clustering markers (**Supplementary Figure S2**).
